# Inhibition of a Mitochondrial Potassium Channel in Combination with Gemcitabine and Abraxane Drastically Reduces Pancreatic Ductal Adenocarcinoma in an Immunocompetent Orthotopic Murine Model

**DOI:** 10.3390/cancers14112618

**Published:** 2022-05-25

**Authors:** Weiwei Li, Gregory C. Wilson, Magdalena Bachmann, Jiang Wang, Andrea Mattarei, Cristina Paradisi, Michael J. Edwards, Ildiko Szabo, Erich Gulbins, Syed A. Ahmad, Sameer H. Patel

**Affiliations:** 1 Department of Surgery, University of Cincinnati, 231 Albert Sabin Way, Cincinnati, OH 45267-0558, USA; li2ww@ucmail.uc.edu (W.L.); wilsong3@ucmail.uc.edu (G.C.W.); magdalena.bachmann@studenti.unipd.it (M.B.); trnnflt@yahoo.com (M.J.E.); gulbineh@ucmail.uc.edu (E.G.); ahmadsy@ucmail.uc.edu (S.A.A.); 2 Department of Biology, University of Padova, Viale G. Colombo 3, 35121 Padova, Italy; ildi@mail.bio.unipd.it; 3 Department of Pathology, University of Cincinnati, 231 Albert Sabin Way, Cincinnati, OH 45267-0558, USA; wajn@ucmail.uc.edu; 4 Department of Pharmaceutical and Pharmacological Sciences, University of Padua, 35122 Padova, Italy; andrea.mattarei@unipd.it; 5 Department of Chemical Sciences, University of Padua, 35122 Padova, Italy; cristina.paradisi@unipd.it; 6 CNR Institute of Neurosciences, University of Padova, Viale G. Colombo 3, 35121 Padova, Italy; 7 Department of Molecular Biology, University Hospital Essen, University of Duisburg-Essen, Hufelandstrasse 55, 45122 Essen, Germany

**Keywords:** pancreas adenocarcinoma, mitochondria, potassium channel, Kv1.3

## Abstract

**Simple Summary:**

Treatment of pancreas ductal adenocarcinoma (PDAC) remains challenging due to the late stage of presentation, limited efficacy of cytotoxic chemotherapies, and aggressive tumor biology. Novel therapeutic targets are desperately needed. The voltage-gated potassium channel, Kv1.3, is one such unique target. It has been extensively studied in many cancers but less is known in pancreas cancer. In this study, we evaluated the tissue expression of Kv1.3 in resected PDAC and tumor inhibition using novel Kv1.3 inhibitors developed by our group (*PCARBTP* and *PAPTP*) with cytotoxic chemotherapies. We found that Kv1.3 is expressed in early stage, non-metastatic, resectable pancreas cancer specimens. Treatment with novel mitochondrial Kv1.3 inhibitors resulted in 95% reduced tumor growth when combined with cytotoxic chemotherapies. This near complete eradication of tumors using this treatment strategy shows that Kv1.3 represents an innovative therapeutic target for pancreas cancer therapy.

**Abstract:**

Pancreas ductal adenocarcinoma (PDAC) is one the most aggressive cancers and associated with very high mortality, requiring the development of novel treatments. The mitochondrial voltage-gated potassium channel, Kv1.3 is emerging as an attractive oncologic target but its function in PDAC is unknown. Here, we evaluated the tissue expression of Kv1.3 in resected PDAC from 55 patients using immunohistochemistry (IHC) and show that all tumors expressed Kv1.3 with 60% of tumor specimens having high Kv1.3 expression. In Pan02 cells, the recently developed mitochondria-targeted Kv1.3 inhibitors *PCARBTP* and *PAPTP* strongly reduced cell survival in vitro. In an orthotopic pancreas tumor model (Pan02 cells injected into C57BL/6 mice) in immune-competent mice, injection of *PAPTP* or *PCARBTP* resulted in tumor reductions of 87% and 70%, respectively. When combined with clinically used Gemcitabine plus Abraxane (albumin-bound paclitaxel), reduction reached 95% and 80% without resultant organ toxicity. Drug-mediated tumor cell death occurred through the p38-MAPK pathway, loss of mitochondrial membrane potential, and oxidative stress. Resistant Pan02 clones to *PCARBTP* escaped cell death through upregulation of the antioxidant system. In contrast, tumor cells did not develop resistance to *PAPTP*. Our data show that Kv1.3 is highly expressed in resected human PDAC and the use of novel mitochondrial Kv1.3 inhibitors combined with cytotoxic chemotherapies might be a novel, effective treatment for PDAC.

## 1. Introduction

Despite the utilization of more aggressive systemic chemotherapy regimens, pancreas ductal adenocarcinoma (PDAC) remains a devastating disease and is the third leading cause of cancer related mortality [1]. In 2022, pancreas cancer is expected to affect approximately 62,210 patients in the US and the incidence will continue to rise. Surgery remains the only option for cure but, unfortunately, only 15–20% of patients are candidates for resection and five-year overall survival remains less than 20% with surgery alone. Apart from surgery and chemotherapy, few effective treatment options exist. Antimetabolites, such as Gemcitabine (GEM) and 5-fluorouracil (5-FU) as well as microtubule depolymerizing agents, such as Nab-paclitaxel (Abraxane), which is a nanoparticle form of albumin-bound paclitaxel are among the most commonly used chemotherapeutic agents [2]. Abraxane depletes tumor stroma, through interaction between albumin and secreted proteins that are acidic and rich in cysteine [3]. However, the survival of patients with PDAC barely reaches one year. Immunotherapy, which has shown dramatic results in many gastrointestinal and cutaneous malignancies, thus far has shown minimal benefit in PDAC. Given these hurdles, novel treatment strategies are desperately needed.

Ion channels are emerging oncological targets, as altered expression and/or function of these druggable proteins [1] is strictly linked to classical cancer hallmarks [4]. Voltage-dependent K^+^ channels (Kv) are a superfamily of ubiquitously expressed membrane proteins that are involved in maintaining membrane resting/action potentials, cell proliferation, immune activation, and cell death [5]. Kv1.3 is a specific voltage-dependent K^+^ channel located mainly in the plasma and inner mitochondrial membranes (mitoKv1.3). First discovered in the plasma membrane of human T lymphocytes, Kv1.3 is also found in tumor and immune cells where it regulates proliferation as well as apoptosis and is aberrantly expressed in malignancies [6,7,8]. Our group has recently developed two specific mitoKv1.3 inhibitors that prevalently and specifically target the mitochondrial channel by virtue of a positively charged triphenylphosphonium group. These inhibitors (*PCARBTP* and *PAPTP*) were shown to selectively kill cancer cells but not normal healthy cells through a reactive oxygen species (ROS)-mediated cell death involving the respiratory chain complex I [9,10,11].

Data in examining Kv1.3 in pancreas cancer are limited and the expression of Kv1.3 in PDAC specimens from cancer patients is unknown. Therefore, in this study we evaluated tissue expression of Kv1.3 in resected human PDAC and found that Kv1.3 is highly expressed. We assessed tumor growth inhibition using the novel inhibitors of mitoKv1.3 alone or in conjunction with cytotoxic chemotherapy in an immune-competent preclinical orthotopic mouse model and provide evidence that the combination therapy drastically reduces tumor size without significant side effects.

## 2. Materials and Methods

### 2.1. Cell Viability Assays

For the cell viability (MTT) assay, Pan02 cells (National Cancer Institute- Frederick Cancer Research and Development Center, Frederick, MD, USA) were seeded in 96-well plates at 0.005–0.01 × 10^6^ cells/well and grown in DMEM + 10% FBS (100 µL/well) for 24 h. In the dark, the growth medium was replaced with a medium containing the desired compound (from a mother solution in DMSO) to the final concentration as shown in the figures. The final DMSO concentration was 0.1% or lower in all cases (including controls). After incubation for 48 h or 24 h for ALDH3A1 knockdown, a CyQUANT™ MTT Cell Viability Assay kit (Fisher, Hampton, NH, USA) was used to detect formazan formation. Formazan formation is a redox-dependent process and can be a confounding factor with ROS build up. MTT reagent was diluted at 1:10 with a culture medium and a 100 µL-well was added into the 96-well plate and cultured for 4 h. 100 µL/well DMSO was added to solve the formazan. Absorbance was measured at 570 nm to detect formazan formation using a Cytation5 plate reader (BioTek, Winooski, VT, USA).

### 2.2. Measurement of Mitochondrial Membrane Potential and ROS Release

To measure mitochondrial membrane potential and ROS levels, Pan02 cells were incubated with 1 µM MitoSOX or 20 nM TMRM in HBSS (Thermo Fisher Scientific, Waltham, MA, USA) at 37 °C for 20 min. After incubation, the compounds were added and the increase in MitoSOX fluorescence or the decrease in TMRM fluorescence was measured by flow cytometry. Median values of the fluorescence intensity distributions (5000 cells were counted) are presented in the data.

### 2.3. In Vitro Development of PCARBTP Resistant Clones

Pan02 cells were detached from tissue culture flasks before reaching confluence by removing the culture medium, adding trypsin-EDTA and incubating for 3 min at 37 °C and 5% CO_2_. After this incubation period, fresh medium was added, and the cells were spun at 200 g for 5 min. Supernatant was removed and fresh medium added. A cell count was carried out with the standard Trypan blue exclusion method. Pan02 cells were seeded at 1 cell per well in 200 µL of fresh medium in 96 flat-bottomed well plates. Clones were inspected regularly so those wells with more than 1 clone could be discarded. The addition of *PCARBTP* was carried out by replacing the medium containing *PCARBTP* at the different doses. If the cells survived at that dose for more than 3 days, we increased the dose, and obtained the 4 clones of Pan02 cells that survived under 10 µM *PCARBTP* in the medium. Resistant cells were amplified under 10 µM of *PCARBTP*, and then the proteins were collected for proteomic analysis.

### 2.4. Proteomic Analysis of Resistant Clones

The Pierce 660 nm Protein assay was performed on a 1:10 dilution of the samples to determine the protein concentration using BSA as a standard. Sufficient protein was present such that 50 µg was taken out to run on a short 1D gel for digestion. A total of 50 µg of each sample (non-resistant and resistant clones) in 40 µL of Laemmli buffer were run 1.5 cm into a 1D 1.5 mm 4–12% BT gel using MOPS running buffer. Pre-stained protein markers were used in surrounding lanes. The regions between the markers and the dye front were excised for trypsin digestion following the standard in gel protocol. The resulting peptides were extracted, dried, and prepared for mass spectrometry. A total of 2.5 µg of each sample was run on the nanoLC-MS/MS in DDA mode and the combined DDA runs were searched using Protein Pilot (SCIEX, AB Sciex Pte. Ltd., Framingham, MA, USA) to create the protein spectral library. A total of 720 proteins were identified with 99% confidence with an FDR of less than 1% at the peptide and protein levels. A matched SWATH-MS method in DIA mode of the samples was used to collect quantitative data for each of the samples for the comparative profiling. Three clones of *PCARBTP* resistant cells and three replicates of normal Pan02 were processed. SWATH-D data analysis workflow was used to validate the data set and detected significant quantitative changes.

### 2.5. Stable Downregulation of ALDH by Lentiviral Transduction

Pan02 cells were grown in a 12-well plate 24 h prior to viral infection. Cells were infected at approximately 50% confluency. To this end, the medium was replaced with medium containing 5 µg/mL of Polybrene^®^ (sc-134220, Santa Cruz, CA, USA) and the cells were infected with 10 µL of ALDH3A1 shRNA (m) lentiviral particles (sc-72033-V, Santa Cruz, CA, USA) in the culture. The plate was swirled gently to mix and incubated overnight. The medium was replaced by a complete medium and cultured for an additional 48 h. Selection of the cells was started at day 5 with 10 µg/mL of puromycin dihydrochloride. The culture medium was replaced by fresh puromycin-containing medium every 3–4 days, until resistant colonies can be identified. Clones were identified, expanded and assayed. Three colonies were chosen, and Western blot analysis was performed to evaluate for ALDH3A1 knockdown (sc-376089, Santa Cruz, CA, USA).

### 2.6. Western Blot

Cells were lysed in whole-cell lysis buffer consisting of 50 mM of HEPES, 150 mM of NaCl, 1 mM of EGTA, 10 mM of sodium pyrophosphatate, 1.5 mM of MgCl_2_, 100 mM of NaF, 10% glycerol and 1% Triton X-100, and pH 7.2, containing an inhibitor cocktail (1 mM of phenylmethylsulfonyl fluoride, 10 mg/mL of aprotinin and 1 mM of sodium orthovanadate) to extract total protein. Protein concentrations were determined using a standard bicinchoninic acid (BCA) assay (23225, Thermo Fisher Scientific, Waltham, MA, USA), and 50 µg of the total protein was subjected to 10% SDS-PAGE followed by electrotransfer onto nitrocellulose membranes. The membranes were blocked in 5% skimmed milk, and then incubated overnight at 4 °C with primary antibodies against human Kv1.3 (1:1000, P4497, Sigma, St. Louis, MO, USA), P-p38 MAPK (1:1000; CST, Framingham, MA, USA), ALDH3A1 (1:1000, sc-376089, Santa Cruz, CA, USA), and β-actin (1:2000; Abcam, Waltham, MA, USA). Membranes were then washed with TBST for 3 × 10 min. This was followed by incubation with horseradish peroxidase-conjugated secondary antibodies for 1 h at room temperature in 5% skimmed milk and washed with TBST for 3 × 10 min. Immunoreactive signals were detected using enhanced chemiluminescence (Pierce, Rockford, IL, USA). Three independent experiments were performed. Original western blots provided in Supplementary Section.

### 2.7. Orthotopic Mouse Pancreatic Tumor Injection Model

All animal experiments were approved by the University of Cincinnati Ethic Committee and Institutional Animal Care and Use Committee. Eight-week-old, wild-type male, C57BL/6J mice were purchased from Jackson Labs (000664, Jackson Labs, Bar Harbor, ME, USA). Mice were anesthetized using 120 mg/kg of ketamine plus 20 mg/kg of xylazine. Orthotopic injection was performed as described by Tepal et al. [12]. In detail, a left subcostal incision was made just below the rib cage and the pancreas was identified. The tumor cell suspension was created by mixing 25 µL of Matrigel with 25 µL of Pan02 cells containing 1 × 10^6^ cells. Pan02 cells were cultured in DMEM + 10% FBS medium, under 37 °C and 5% CO_2_, with no antibiotic added. The tumor suspension was slowly injected into the pancreas and the needle was left in place for 60 s to allow the Matrigel to set. After ensuring hemostasis, the abdomen was closed in 2 layers using 3-0 silk sutures.

### 2.8. In Vivo Kv1.3 Inhibitor and Cytotoxic Chemotherapy Administration

*PCARBTP* was suspended in 50% DMSO and injected into the peritoneal cavity at a dose of 15 nmol/gbw on day 6 after tumor injection. Similarly, *PAPTP* was administrated at a dose of 5 nmol/gbw. Gemcitabine was dissolved in ddH_2_O and injected into the intraperitoneal cavity at dose of 190 nmol/gbw 6 days after tumor injection. Abraxane was dissolved in DMSO and intraperitoneally injected at a dose of 23.4 nmol/gbw. Tumor volume was calculated using the formula: volume = length × width × depth.

### 2.9. Immunohistochemistry

Immunohistochemistry (IHC) staining was performed using a biotin-streptavidin-peroxidase (SP) kit (AB64269, Abcam, Waltham, MA, USA) and a diaminobenzidine kit (DAB) as previously described [13]. Institutional Review Board approval was obtained to obtain human resected pancreas ductal adenocarcinoma specimens and associated clinicopathologic data (IRB 2019-0324). Tumor specimens were obtained from the University of Cincinnati Department of Pathology. Five-micrometer sections were deparaffinized and rehydrated in xylene and gradients of ethanol. Slides were boiled in citrate buffer (10 mM of sodium citrate, 10 mM of citric acid, pH 6.0) at 92–98 °C for 10 min to retrieve the antigen. The sections were then incubated with 3% H_2_O_2_ in methanol for 10 min to quench endogenous peroxidase and blocked with normal goat serum for 20 min. Sections were incubated with specific primary antibodies against Kv1.3 (1:200; P4497, Sigma, MO, USA) at 4 °C overnight. The sections were then incubated with biotinylated goat-anti-rabbit IgG secondary antibody and stained with DAB working reagent (per manufacturer’s instructions) for 30–60 s. They were then counterstained with hematoxylin. Finally, sections were mounted with Permount (SP15-500, Thermo Fisher Scientific, Waltham, MA, USA) onto slides. Negative control was performed using unconjugated rabbit IgG (011-000-003, Jackson ImmunoResearch Laboratories, West Grove, PA, USA). Images were acquired with a ZEISS AXIO microscope (Carl ZEISS, Jena, Germany). Slides were scored by a gastrointestinal pathologist who specializes in evaluating pancreas cancer. He was blinded to all clinicopathologic data and determined the percent and intensity of staining (scored 0–3). A final score of high versus low expression was determined if patients had >70% of tumor cells staining with a score of 3 for intensity. The remaining group was classified as low.

### 2.10. Hematoxylin & Eosin Staining

Slides containing paraffin sections were passed using the following steps: 3 × 5 min in Xylene (blot excess xylene before going into ethanol), 2 × 5 min in 100% ethanol, 1 × 5 min in 95% ethanol, 1 × 5 min in 70% ethanol, 1 × 5 min deionized H_2_O, and 1 × 3 min Hematoxylin. They were then rinsed with deionized water 1 × 5 min, tap water and ethanol to destain. After subsequent rinse, they were treated for 1 × 30 sec with Eosin, for 3 × 5 min with 95% ethanol, for 3 × 5 min with 100% ethanol, for 3 × 5 min with Xylene, and a coverslip was placed using Permount mounting medium (SP15-500, Thermo Fisher Scientific, Waltham, MA, USA).

### 2.11. TUNEL Assay

Animals were sacrificed and immediately perfused via the right heart with 0.9% NaCl for 2 min followed by 10% formalin for 10 min. Organs, including the heart, lung, liver and kidney, were then removed and further fixed in 10% formalin for 48 h. TUNEL staining was performed with an In Situ Cell Death Detection Kit as instruction by the supplier. Briefly, the tissues were dehydrated, embedded in paraffin, and sectioned at a thickness of 5 µm. Sections were then dewaxed, rehydrated and incubated for 5 min in 0.1 M citrate buffer (pH 6.0) at 350 W in a microwave. After this, samples were immediately cooled in PBS and incubated with TMR coupled dUTP in the presence of terminal deoxynucleotidyl-transferase (Roche, Basel, Switzerland) at 37 °C for 60 min. Samples were embedded with mounting medium with DAPI prior to analysis. An excitation wavelength of 488 nm was used and evaluated using a ZEISS AXIO microscope (Carl ZEISS, Jena, Germany).

### 2.12. Statistical Analysis

The tumor volume and mass and mouse body weight were analyzed by *t*-test and one-way ANOVA using GraphPad Prism 9.0, each experiment has more than 6 mice in 1 group, indicated in Results and Figure legends. *p* < 0.05 was considered as statistically significant. * *p* < 0.05; ** *p* < 0.01, *** *p* < 0.0005, **** *p* < 0.0001.

### 2.13. Immunofluoresence

Immunofluorescence staining was performed using Pan02 cells. These were seeded on a coverslip at 50% confluency 12 h before staining. Cells were washed with cold PBS × 3 then fixed by 4% paraformaldehyde for 30 min. Cells were again rinsed with cold PBS × 3, followed by 1% Triton-100 for 30 min. Cells were blocked with 5% donkey serum for 1 h at room temperature and washed with cold PBS for 10 min × 3. Primary antibodies Kv1.3 (1:100, APC-101, Alomone labs, Jerusalem, Israel) and TOM20 (1:100, MABT166, Sigma, Burlington, MA, USA) were incubated overnight at 4 °C. Cells were washed with cold PBS for 10 min × 3, secondary antibodies goat anti-mouse antibody Alexa 488 (1:2000, A11001, Thermo Fisher Scientific, Waltham, MA, USA) and goat anti-rabbit antibody Alexa Fluor™ 594 (1:2000, A11012, Thermo Fisher Scientific, Waltham, MA, USA) were incubated for 1 h at room temperature. Cells were washed with cold PBS for 10 min × 3 and mounting medium with DAPI was added. Carl Zeiss LSM 710 confocal laser-scanning microscope (Carl Zeiss, Jena, Germany) was used to capture the images.

## 3. Results

### 3.1. Kv1.3 Is Highly Expressed in Resected Human Specimens

**Figure 1:**
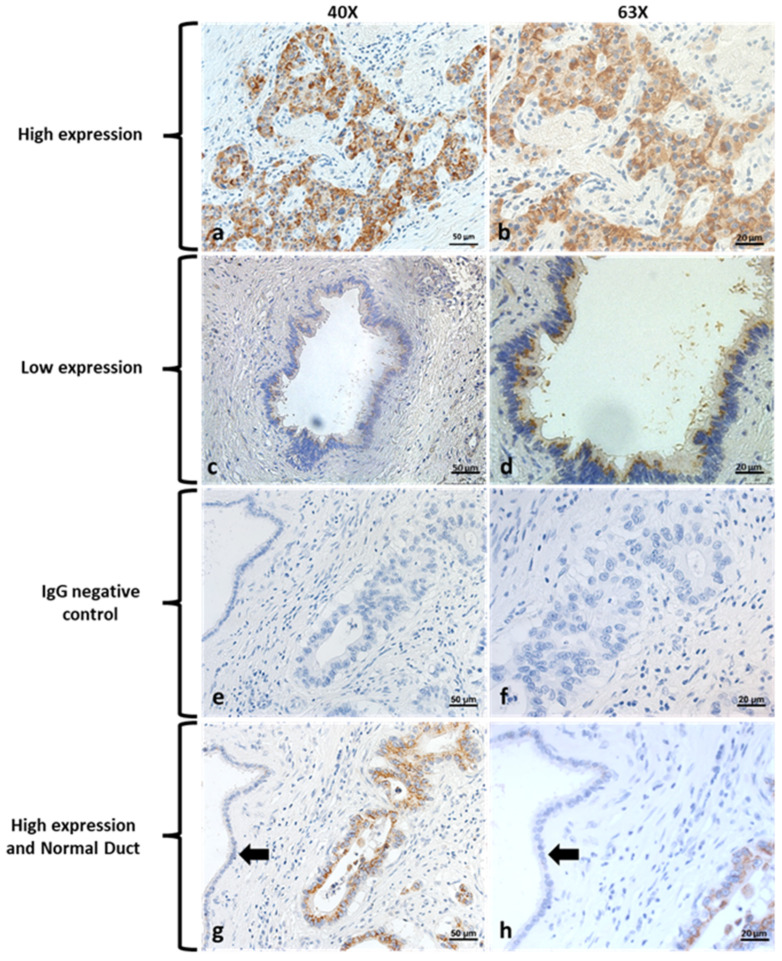
Expression of Kv1.3 in human pancreatic cancer tissue. Expression of Kv1.3 in human pancreatic tissue. Panel (**a**), high expression level of Kv1.3 in human pancreatic cancer tissue. (*n* = 33 out of 55, scale bar = 50 µm). Panel (**b**), magnification of panel (**a**), scale bar = 20 µm. Panel (**c**), low expression level of Kv1.3 in human pancreatic cancer tissue. (*n* = 22 out of 55, scale bar = 50 µm). Panel (**d**), magnification of panel c, scale bar = 20 µm. Panel (**e**), Immunoglobulin G (IgG) control with magnification (Panel (**f**)). Panel (**g**) shows a pancreas adenocarcinoma ductule with high expression of Kv1.3 (brown color) and adjacent normal pancreas parenchyma without Kv1.3 expression (black arrow), scale bar = 50 µm. Panel (**h**), magnification of panel (**g**), scale bar = 20 µm.

As Kv1.3 is emerging as a promising oncological target [14], limited information is available about expression of this channel in human PDAC tissues. First, we assessed Kv1.3 expression by immunohistochemistry (IHC) evaluation on tissues from 55 patients who were diagnosed at a relatively early stage and underwent resection for PDAC (Figure 1). Median patient age was 68 years old with 42% of patients being female, 86% white, and 9% black. A pancreaticoduodenectomy was performed in 69.1% of patients, distal pancreatectomy in 29.1%, and total pancreatectomy in 1.8%. On final pathology, 29.1% of specimens had poorly differentiated tumors, 90.9% had perineural invasion (PNI), and 56.4% lymphovascular invasion (LVI). Positive lymph nodes were found in 83.6% of patients. American Joint Committee on Cancer (8th edition) staging showed 52.7% were stage 2, 34.5% stage 3, and 12.7% stage 1.

Kv1.3 IHC staining showed that all pancreas tumor specimens exhibited expression of Kv1.3. Based on percentage and intensity of stain, we found that 60.0% of tumor specimens had high expression (*n* = 33). Only 8.3% of normal pancreas specimens from the same surgical specimens had expression of Kv1.3. Over a median follow up of 28.0 months, 67.9% of patients developed a recurrence and 65.5% of patients died. When stratified by Kv1.3 expression, median recurrence free survival was 16 months with high Kv1.3 expression versus 17 months with low expression (*p* = 0.36) (Figure S1).

Median overall survival was 27 months with high Kv1.3 expression and did not reach 30 months for low Kv1.3 expression (*p* = 0.53) (Figure S1).

High expression of Kv1.3 in tumor specimens was not associated with having a positive lymph node, poor differentiation, perineural invasion, or lymphovascular invasion (*p* > 0.05). These data suggest that Kv1.3-expression neither determines prognosis of the tumor per se nor tumor metastasis. We thus investigated whether Kv1.3 might serve as a novel target to treat PDAC.

### 3.2. PAPTP and PCARBTP Trigger Apoptosis in Pan02 Cells by Enhancing Mitochondrial ROS Production and Inducing Loss of Membrane Potential

**Figure 2:**
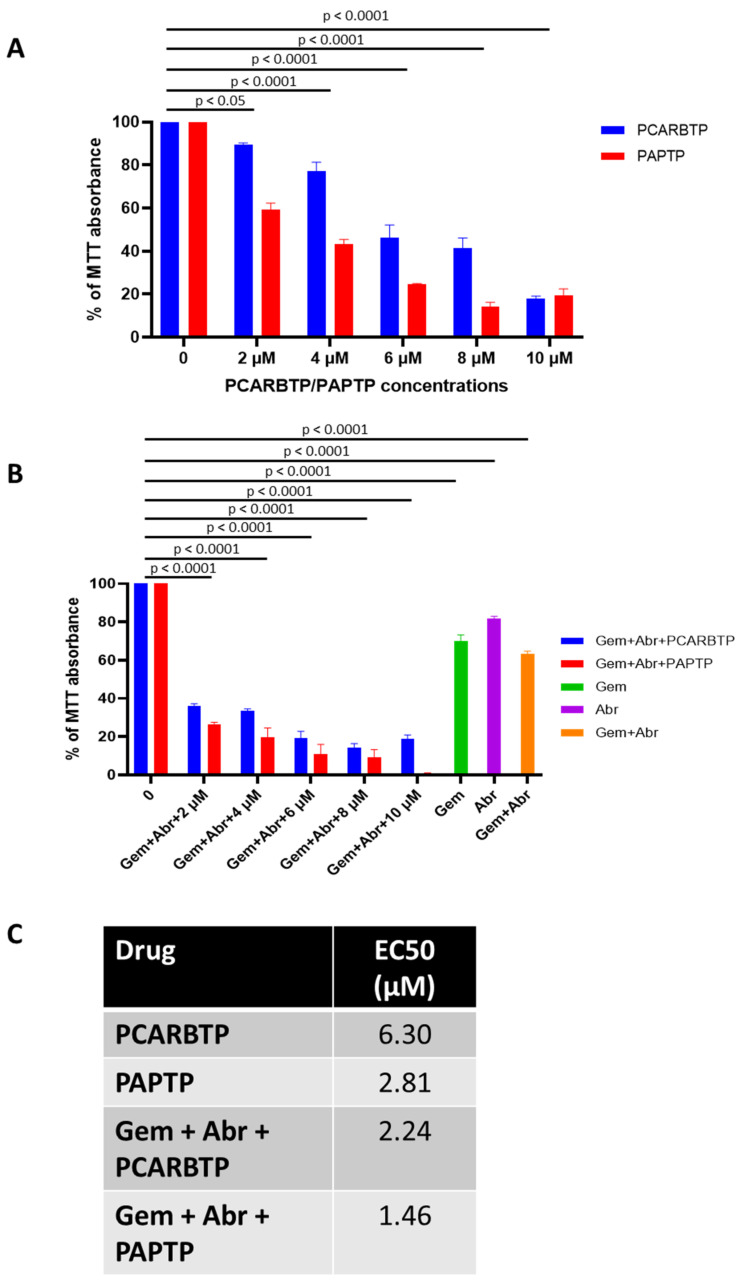
PCARBTP and PAPTP trigger apoptosis in Pan02 cells by enhancing mitochondrial ROS product and combination with Gemcitabine and Abraxane enhances tumor cell death. (**A**) MTT assay performed on Pan02 cells treated with *PCARBTP*/*PAPTP* or (**B**) combined with Gemcitabine and Abraxane as indicated (*n* = 3, mean ± SD). (**C**) EC50 of *PCARBTP* and *PAPTP* alone and in combination Gemcitabine and Abraxane.

To test the significance of Kv1.3 expression in pancreas cancer cells, we treated the widely used Pan02 mouse PDAC line with *PAPTP* and *PCARBTP*, two inhibitors of mitochondrial Kv1.3. Pan02 tumor cells harbor no mutations in KRAS, Cdkn2a, or Tp53 and have a high resistance to a wide range of chemotherapeutic agents [15]. Supplementary Figure S2 shows expression of Kv1.3 in wild type Pan02 cells and localization to the mitochondria (Figure S3). MTT assay was performed on Pan02 cells treated with different concentrations of *PCARBTP and PAPTP* for 48 h, revealing *PCARBTP* killed Pan02 cells by a dose-dependent way (Figure 2A).

### 3.3. Combination with Gemcitabine and Abraxane Enhances Tumor Cell Death Obtained by PAPTP or PCARBTP Treatment

Gemcitabine and Abraxane have recently been used together in the MPACT clinical trial which showed benefit in terms of overall survival. As the EC50 of cell survival is around 0.3 µM for GEM in Pan02 cells [16], we used a combination of 0.25 µM GEM with 20 nM Abraxane and various concentrations of *PAPTP*/*PCARBTP*. 8 µM *PCARBTP* and 10 µM of *PAPTP* with 0.25 µM of Gemcitabine and 20 nM of Abraxane resulted in the highest rate of tumor cell death of 85.8% and 99.2%, respectively (Figure 2B). Interestingly, while the combination treatment with PCARBTP reduced survival of Pan02 cells by more than 80% at the higher concentrations, at 10 µM concentration *PAPTP* + GEM + Abraxane reduced cell survival to zero when continuous treatments lasted for 48 h. EC50 values were obtained for the different combination treatments above (Figure 2C). The combination index was <1 according to Compusyn, indicating a synergistic effect between *PAPTP*/*PCARBTP* and Abraxane plus gemcitabine.

### 3.4. PCARBTP and PAPTP Reduced Pancreatic Ductal Adenocarcinoma Tumor Size in a Syngeneic Orthotopic Mouse Model

Following the above in vitro experiments, we tested the effects of PAPTP/PCARBTP either alone or in combination with GEM + Abraxane in orthotopic models using Pan02 cells, which are known to give rise to well-differentiated tumors with high mutational burden [15]. Since no studies have been performed on PDAC in immune-competent mice with mitoKv1.3 inhibitors up to now, tumor growth was examined in a syngeneic model using Pan02 cells.

**Figure 3:**
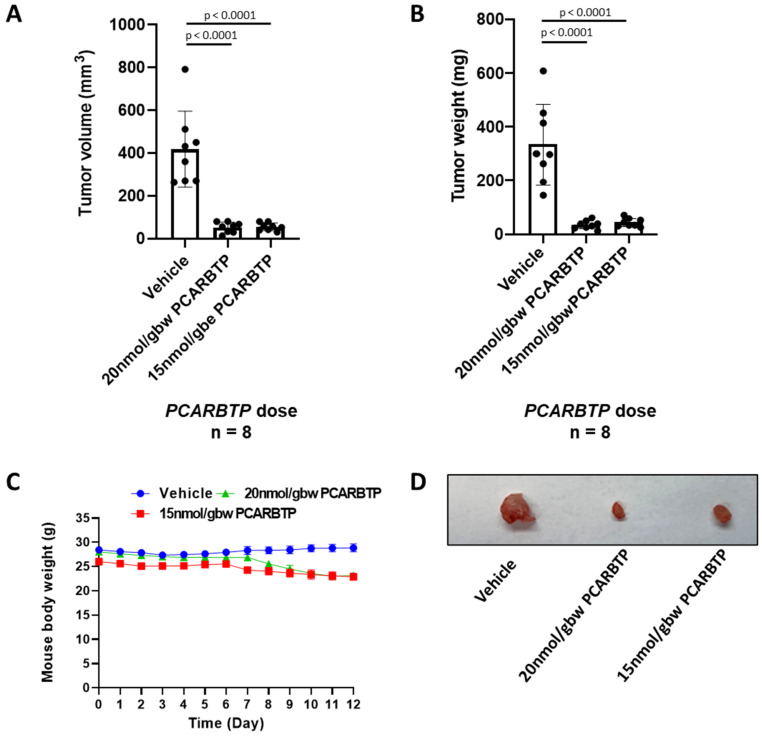
*PCARBTP* reduced pancreatic tumor size in an orthotopic mouse model. (**A**,**B**) *PCARBTP* reduced mouse pancreatic tumor volume (**A**) and weight (**B**) orthotopically significantly upon intraperitoneal administration, *n* = 8. (**C**) Body weights change in the mice during *PCARBTP* treatment were less than 20% (*p* < 0.05). (**D**) Representative pictures of the mouse orthotopically injected tumors.

Intraperitoneal injection of *PCARBTP* and *PAPTP* was performed 6 days after tumor injection, and tumors were collected 12 days after. Non-toxic [9] doses of 15 nmol/g and 20 nmol/g body weight of *PCARBTP* were injected every other day for 3 doses. Likewise, 5 nmol/g body weight *PAPTP* was injected every other day for 3 doses. The tumor volume and mass treated with 15 nmol/gbw *PCARBTP* were reduced by 87% and 87% (*n* = 8), while reduction of the tumor volume and mass treated with 20 nmol/gbw *PCARBTP* reached 88% and 90%, respectively (*n* = 8) (Figure 3A,B). Although bodyweight slightly decreased with respect to pre-orthotopic weight after *PCARBTP* treatment, no mice had greater than a 20% decrease (Figure 3C). Representative images are shown in Figure 3D.

**Figure 4:**
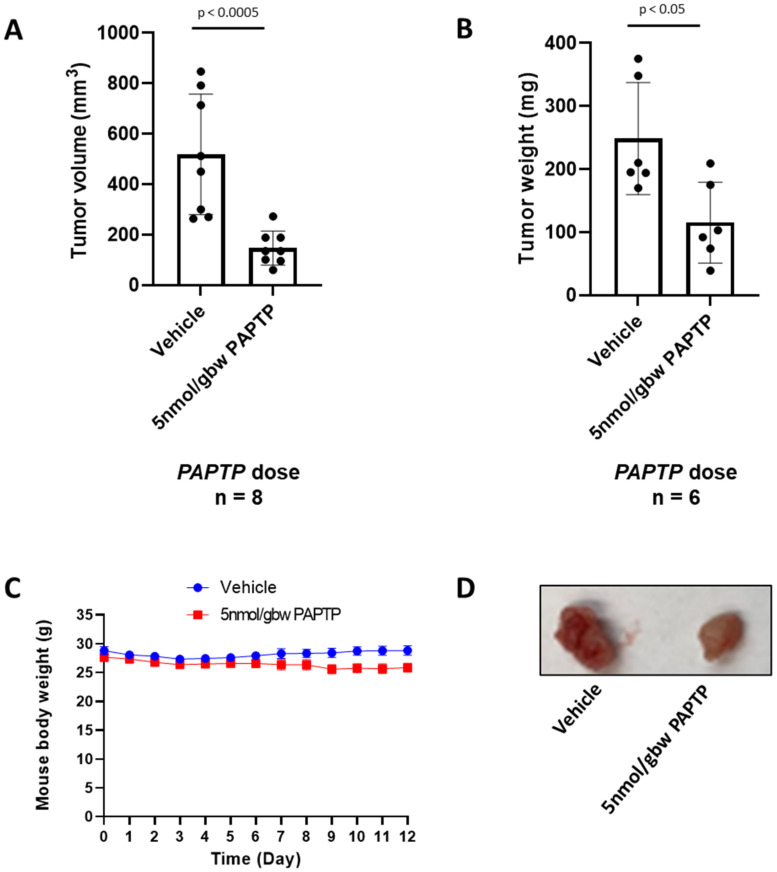
*PAPTP* reduced pancreatic tumor size in an orthotopic mouse model. (**A**,**B**) *PAPTP* reduced mouse pancreatic tumor volume (**A**) and weight (**B**) orthotopically significantly by intraperitoneal administration, *n* = 6. (**C**) Body weights change of the mice during *PAPTP* treatment were not significantly changed (*p* = 0.17). (**D**) Representative pictures of the mouse orthotopically injected tumors.

*PAPTP* was less effective in this model: the tumor volume and mass treated with 5 nmol/gbw of *PAPTP* were reduced by 70% (*n* = 6) and 64% (*n* = 6), respectively (Figure 4A,B). There were no significant changes in the body weights of the mice with *PAPTP* treatment, Figure 4C (*p* = 0.17). Representative tumors are shown in Figure 4D.

### 3.5. Gemcitabine-Abraxane Treatment with PCARBTP/PAPTP Drastically Reduced Tumor Growth

**Figure 5:**
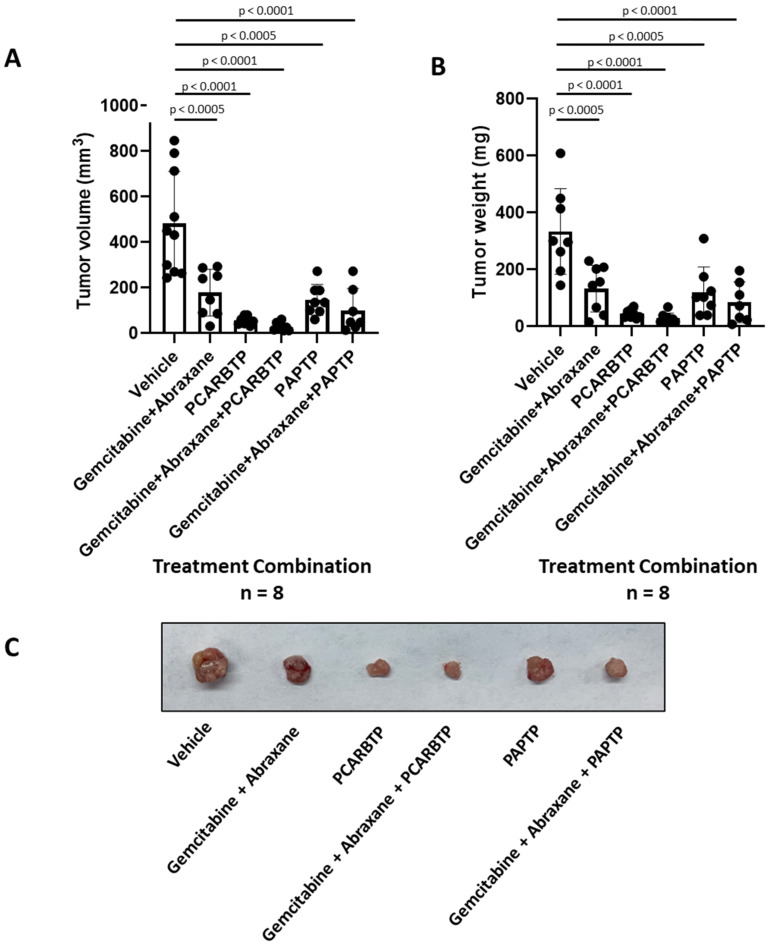

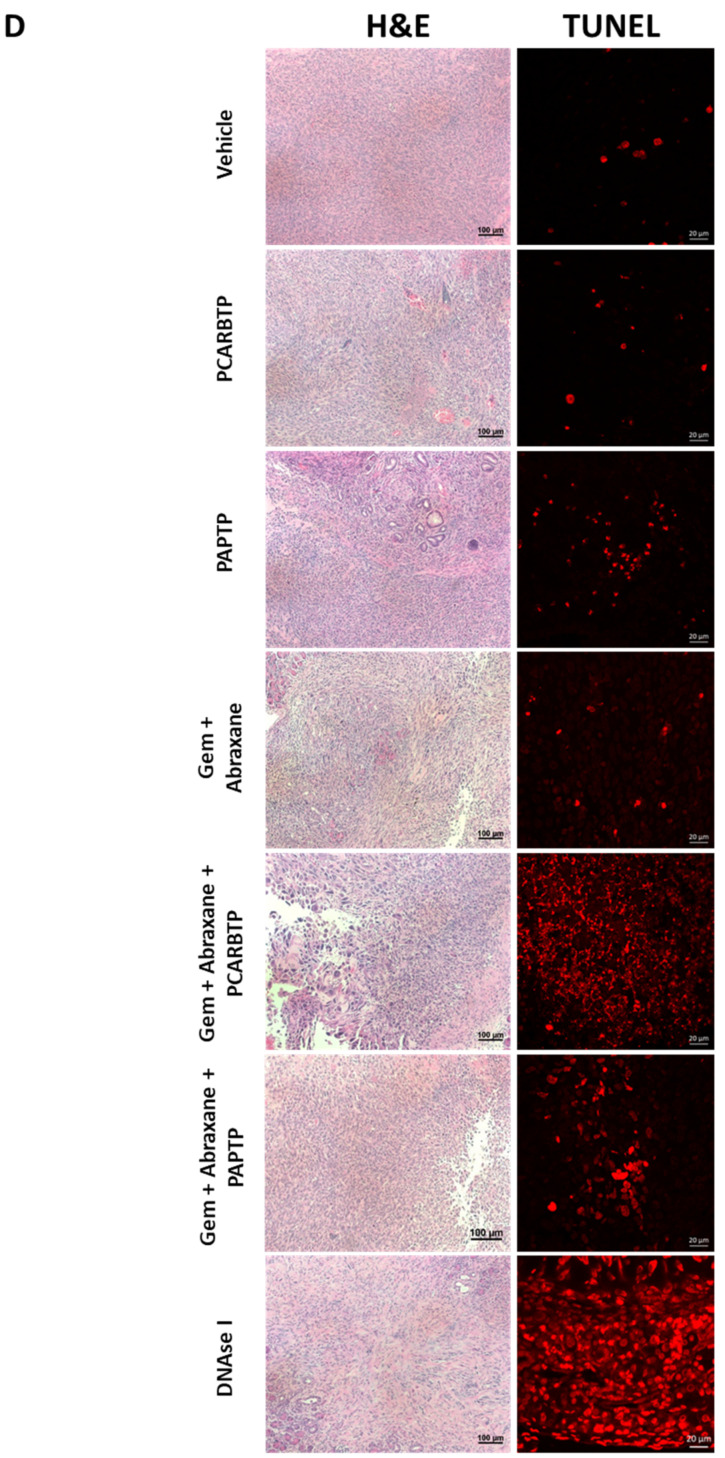
*PCARBTP* or *PAPTP* combined with Gemcitabine and Abraxane drastically reduced pancreatic tumor size and weight an orthotopic mouse model. (**A**,**B**) *PCARBTP* and *PAPTP* combined with Gemcitabine and Abraxane reduced up to 95% the pancreas tumor volume (**A**) and weight (**B**) compared to the control group. *n* = 8. (**C**) Representative pictures of the mouse orthotopically injected tumors. (**D**) H&E staining and TUNEL assay of indicated organs. DNase treatment was used as a positive control. Treatment groups shown in in the figure resulted in greater tumor cell death compared to the vehicle control. Scale bars for H&E = 100 µm, scale bars for TUNEL = 20 µm.

We then examined if there was a synergistic effect of using mitoKv1.3 inhibitors with cytotoxic chemotherapies already used in the clinical practice, given our in vitro results shown in Figure 2. Compared to untreated controls, 190 nmol/gbw Gemcitabine plus 23.4 nmol/gbw Abraxane reduced the tumor volume and mass by only 64% and 60% (*n* = 8), respectively. The concentration of GEM was chosen on the basis of the literature where Pan02 cells were used (e.g., [16,17,18]). In contrast, the combination of Gemcitabine with Abraxane plus 15 nmol/gbw *PCARBTP* massively reduced the tumor volume and mass by 95% and 92%, respectively (*n* = 8). Similarly, we found a substantial tumor volume and mass reduction after treatment with Gemcitabine, Abraxane, and 5 nmol/gbw *PAPTP* of 80% and 75% (Figure 5A–C). H&E staining and TUNEL assay were performed to detect the apoptotic level of tumor cells, by sectioning through the tumor (Figure 5D). Most of the tumor cells were dying through apoptosis with the Gemcitabine, Abraxane plus *PCARBTP* treatment.

### 3.6. Combined Treatment with Mitochondrial Kv1.3 Inhibitors and Cytotoxic Chemotherapies Had No End Organ Toxicities

**Figure 6:**
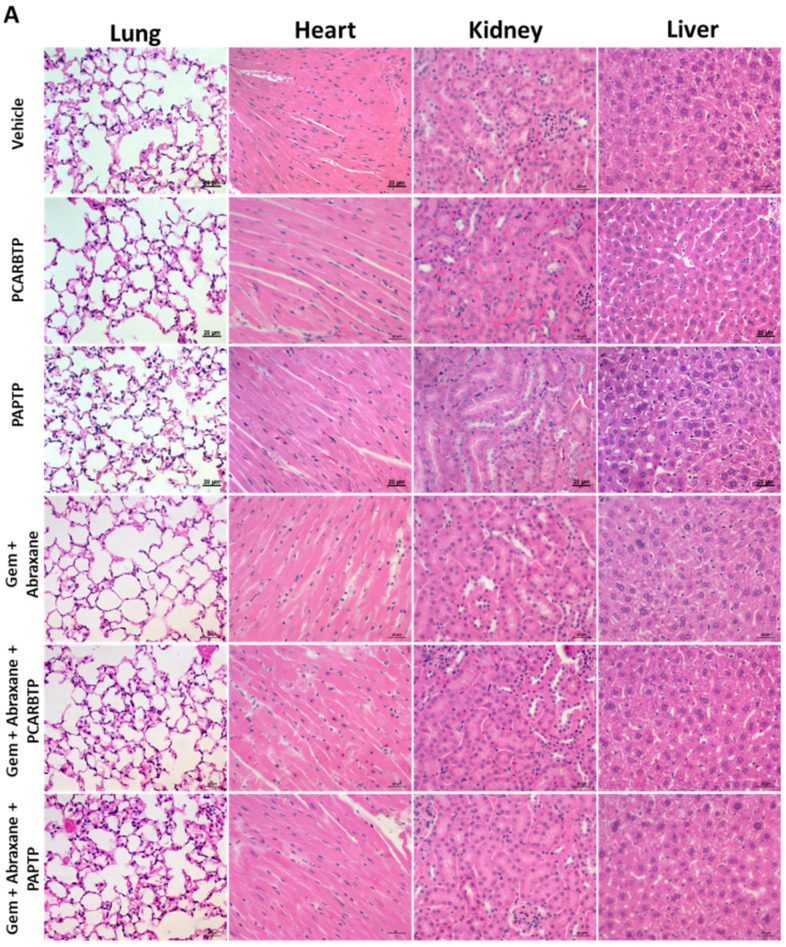

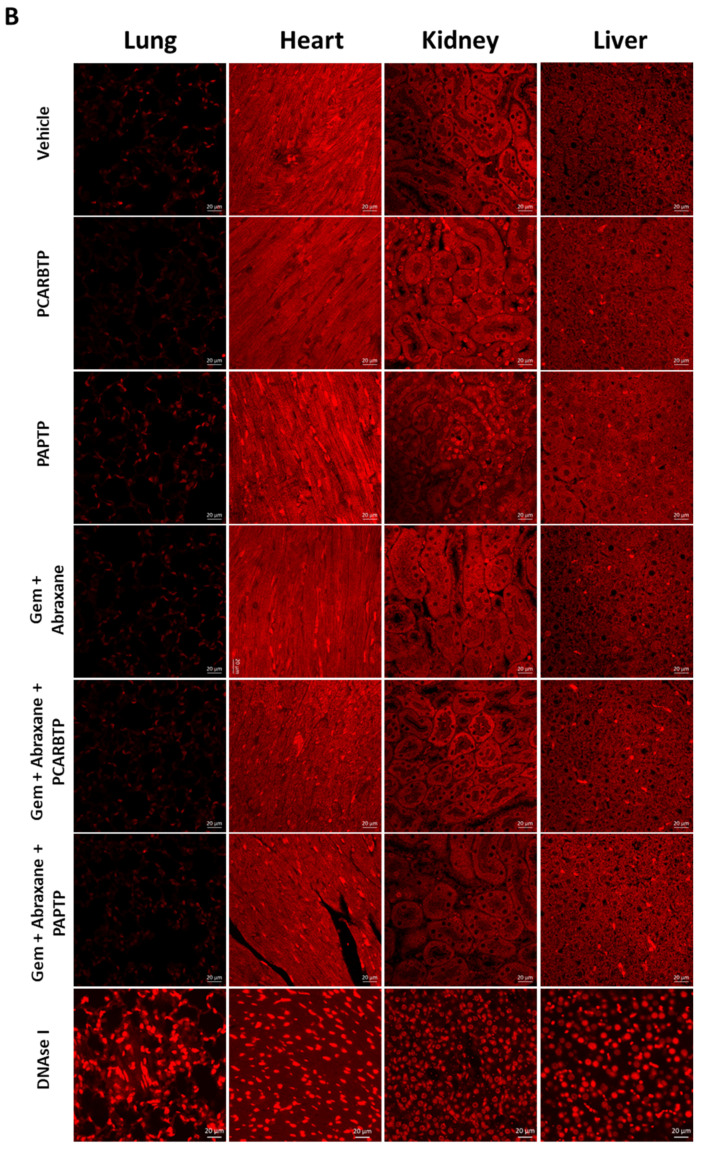
Lack of *PCARBTP/PAPTP* toxicity on other organs in a mouse model. (**A**) H&E staining showed either *PCARBTP* or *PAPTP* intraperitoneal administration alone or combined with Gemcitabine and Abraxane had no toxic effect on the heart, lung, kidney, or liver. Scale bar = 20 µm. (**B**) TUNEL assay performed on the respective organs. DNase treatment was used as a positive control. There was lack of toxicity in the lung, heart, kidney and liver when treated with *PCARBTP or PAPTP* alone or when combined with cytotoxic chemotherapies. Scale bars, 20 µm.

These data indicate a marked effect of the combination of *PCARBTP/PAPTP* with Gemcitabine/Abraxane on pancreas carcinoma in vivo. Importantly, we observed that mice did not show signs of distress and H&E staining at the time of sacrifice after treatment with the combination of *PCARBTP/PAPTP* with Gemcitabine/Abraxane (see concentrations above) showed there was no significant toxicity to the heart, lung, liver, and kidney (Figure 6A). TUNEL assay showed the lack of inhibitor-induced apoptosis in healthy tissues (Figure 6B). These results indicate a specific effect of the treatment on the malignant tumor.

### 3.7. Mitochondrial Kv1.3 Inhibitor PCARBTP Treatment Activated Phosphorylation of p38 MAPK In Vitro and In Vivo

**Figure 7:**
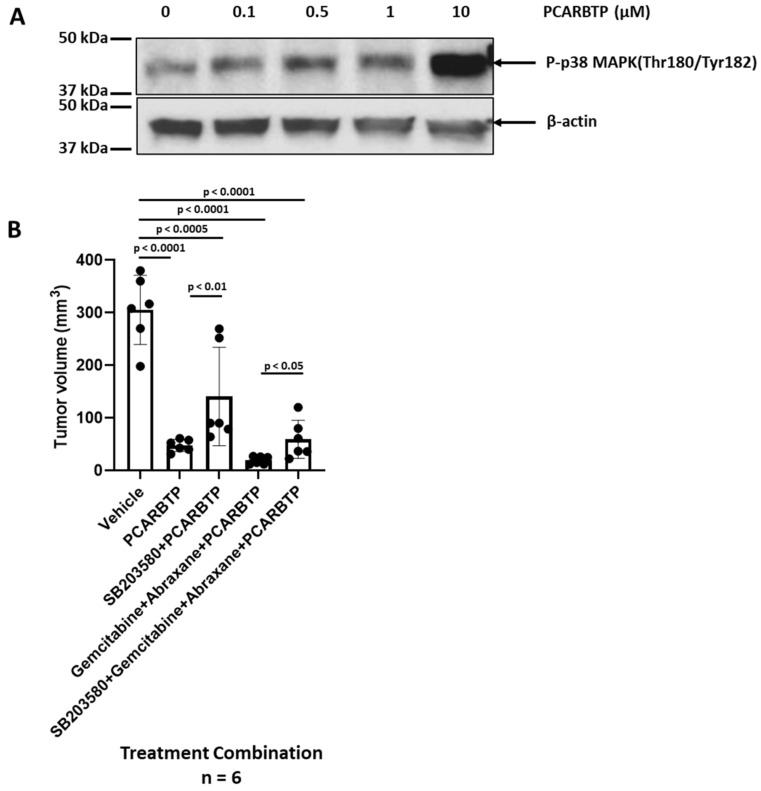
*PCARBTP* mediated cell apoptosis through the p38 MAPK pathway. (**A**) Phosphorylation levels of p38 MAPK (Thr180/Tyr182) increased with *PCARBTP* treatment for 30 min in a dose-dependent manner, *n* = 3. (**B**) p38 MAPK inhibitor (SB203580) attenuated the function of *PCARBTP* or *PCARBTP* combined with Gemcitabine and Abraxane, *n* = 6.

We have previously shown that the action of sub-lethal concentrations of *PCARBTP* was exacerbated when the stress response kinase JNK was inhibited in Jurkat lymphocytes [19]. Here, we evaluated the effect of various concentrations of *PCARBTP* on activation of the other major stress-activated signaling kinase, namely p38 mitogen-activated protein kinase (MAPK), given that this kinase regulates apoptosis also in PDAC lines (e.g., [13]). In vitro, Pan02 cells were treated with *PCARBTP* (up to 10 µM) for 30 min. Western blot analysis highlighted an increase in phosphorylation level of this p38MAPK when treating the cells with relatively high concentration of the drugs that were able to induce mitochondria ROS production within the same timeframe (Figure 7A). GEM was also reported to activate c-JUN N-terminal kinase (JNK) and p38 mitogen activated protein kinase (p38 MAPK) in PDAC lines and GEM-induced JNK and p38 MAPK activation mediated increased apoptosis [13].

Thus, to test the significance of p38 MAPK for mitoKv1.3 inhibitor- (and triple treatment) mediated cell death, we examined the effects of treatment with the p38 MAPK inhibitor, SB203580. A total of 13 nmol/gbw of SB203580 (a dosage used in other in vivo studies, e.g., [20]) was found to attenuate the effects of *PCARBTP* in an orthotopic mouse model (Figure 7B). Compared to the control, tumor volume from all drug-treated groups were significantly decreased (*p* < 0.05). Compared to *PCARBTP* only, addition of SB203580 to *PCARBTP* attenuated growth inhibition by mitoKv1.3 inhibitor treatment (tumor volume 87% vs. 51%, *p* < 0.01). Similarly, the addition of SB203580 to Gemcitabine–Abraxane–*PCARBTP* attenuated the tumor-reducing effect of the drugs, as tumor volume was reduced only by 80% (versus 95%) in the presence of p38 MAPK inhibitor (*p* < 0.05), suggesting that *PCARBTP* mediated cell death occurs at least in part through the p38 MAPK pathway in vivo. Supplementary Figure S4 shows antibody specificity of P-p38 MAPK in Pan02 cells.

**Figure 8:**
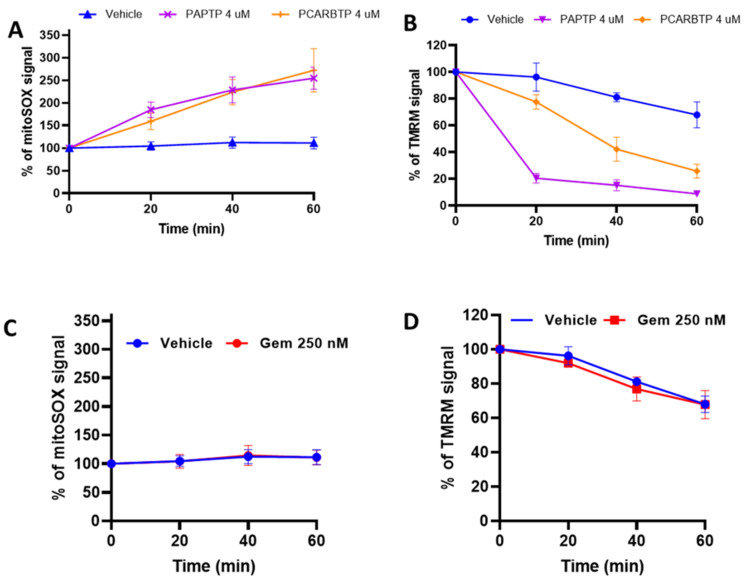
Direct effects of *PCARBTP/PAPTP* alone or when combined with Gemcitabine (GEM) and Abraxane on Mitochondria. (**A**) Mitochondrial ROS production was tested using MitoSOX on Pan02 cells treated with *PCARBTP/PAPTP*. The compounds increased ROS level. Mean normalized values ± SD from four biological replicates are shown. (**B**) Mitochondrial membrane potential was followed using 5 nM TMRM on Pan02 cells treated with *PCARBTP/PAPTP* for the indicated time. In these experiments, the probe was not washed off so as to allow further uptake following hyperpolarization. Results are shown as mean ± SD from four biological replicates. (**C**) Mitochondrial ROS production on Pan02 cells treated with GEM. (**D**) Mitochondrial membrane potential on Pan02 cells treated with GEM.

To identify further signaling pathways that mediate the effects of *PCARBTP* and *PAPTP* on pancreas cancer cells, mitochondrial ROS production was tested using the fluorogenic dye MitoSOX, which correlates with mitochondrial superoxide production. Mitochondrial membrane potential changes were followed by the membrane potential-sensitive dye tetramethylrhodamine methyl ester (TMRM). *PCARBTP* or *PAPTP* both induced an increase in the MitoSOX fluorescence signal (Figure 8A) and mitochondrial depolarization (Figure 8B), consistent with previous data [9]. Figure S5 shows histograms of flow cytometry from mitoSOX and TMRM analyses.

Gemcitabine might synergistically act with mitoKv1.3 inhibitors by further enhancing cellular ROS level, as GEM has been shown to trigger ROS production in PDAC cells via indirect activation of NAD(P)H oxidase (NOX) [21] (through activation of NF-κB). However, GEM when applied alone, did not cause changes in mitochondrial ROS production (Figure 8C) and of membrane potential (Figure 8D). The same observation is true for Abraxane. A combination of the three drugs did not enhance further the effect of PAPTP/PCARBTP on mitochondrial parameters.

### 3.8. Resistance to PCARBTP Treatment

**Figure 9:**
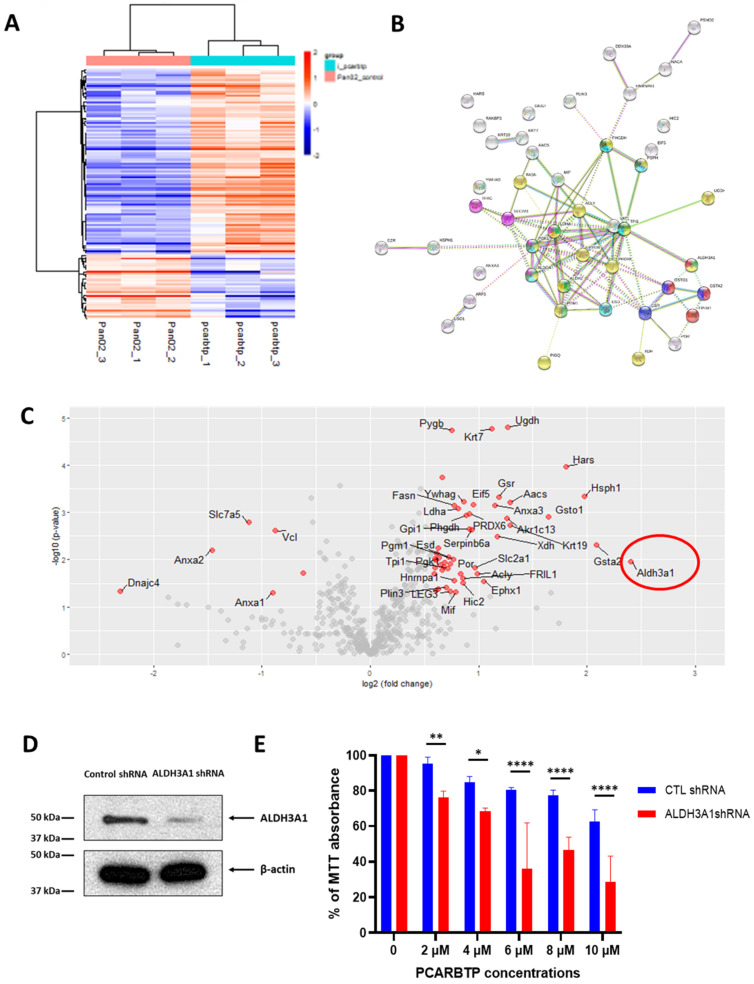

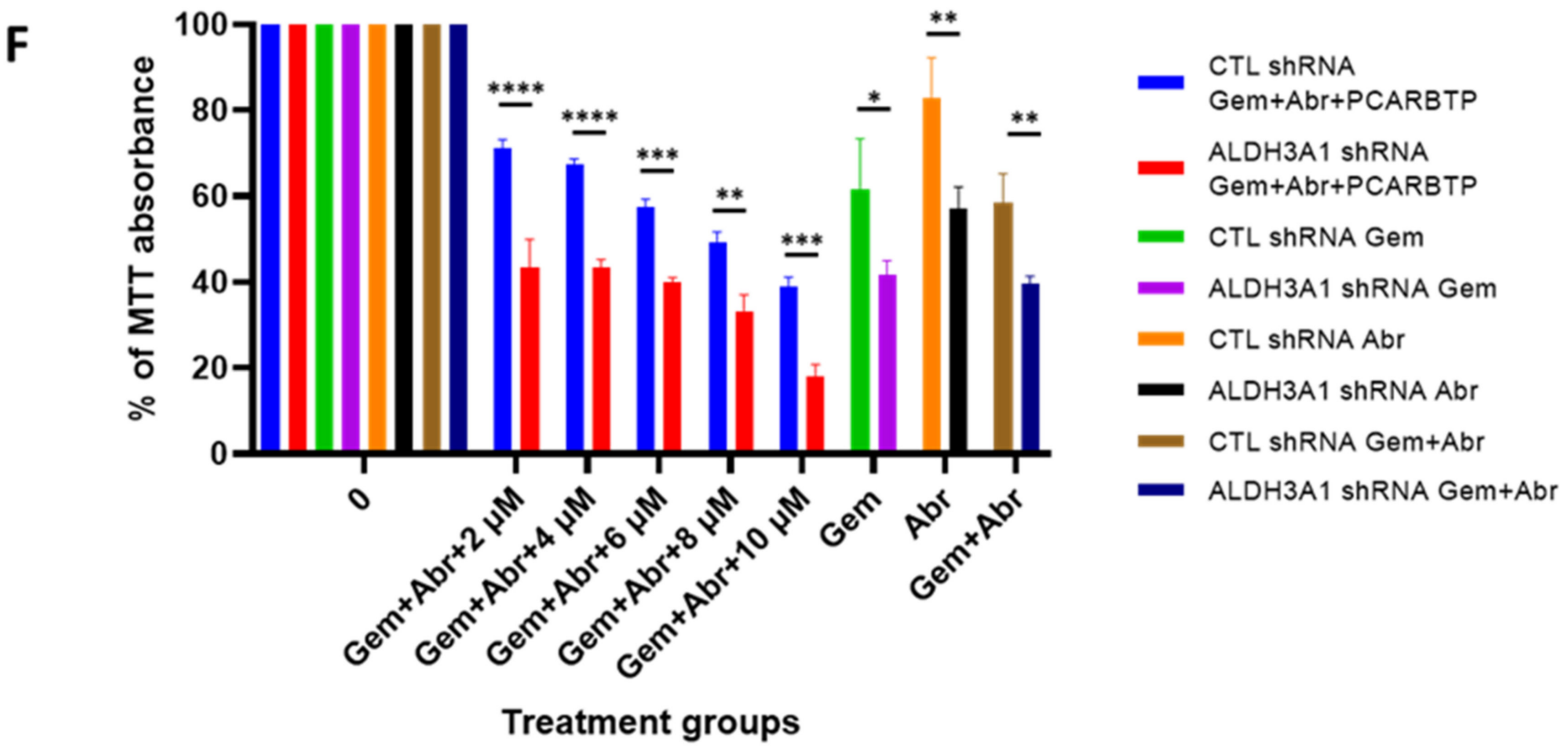
Drug resistance to PCARBTP in cultured Pan02 cells. (**A**) Proteomic analysis of protein changes in PCARBTP resistant clones. Heatmap of unbiased clustering of only the significant proteins, 135 proteins with ANOVA *p* < 0.05 (color in Log2-scale), *n* = 3. (**B**) STRING analysis showing the following enriched KEGG pathway-linked proteins. Number of nodes: 45; number of edges: 89; average node degree: 3.96; avg. local clustering coefficient: 0.521; expected number of edges: 23; PPI enrichment *p*-value < 1.0 × 10^−16^. KEGG pathways: light green: Glycolysis/Gluconeogenesis (hsa00010) (*p* value 3.45 × 10^−8^); yellow: metabolic pathways (hsa01100) (*p* value 4.26 × 10^−7^); light blue: carbon metabolism (hsa01200) (*p* value 1.08 × 10^−5^); dark green biosynthesis of amino acids (hsa01230) (*p* value 2.10 × 10^−5^); magenta: HIF-1 signaling pathway (hsa04066) (*p* value 7.12 × 10^−5^); red: metabolism of xenobiotics by cytochrome P450 (hsa00980) (*p* value 0.00039); dark blue: glutathione metabolism (hsa00480) (*p* value 0.0029). (**C**) Volcano plot analysis for significant up/down regulated proteins (PCARBTP/Pan02_control), ALDH3A1 in red circle is the most upregulated protein. (**D**) Western blot showed ALDH3A1 was knocked down by ALDH3A1 lentiviral shRNA. (**E**,**F**) MTT assay performed on Pan02 cells transfected with control shRNA and ALDH3A1 shRNA then treated with PCARBTP/PAPTP or combined with 0.25 µM of Gemcitabine and 20 nM of Abraxane as indicated (*n* = 3, mean ± SD), * *p* < 0.05; ** *p* < 0.01, *** *p* < 0.0005, **** *p* < 0.0001.

Given the very promising in vivo effects observed with the mitoKv1.3 inhibitors, we aimed at understanding whether, similarly to GEM [21], resistance to *PCARBTP* and *PAPTP* may occur on a longer timescale. To this end, we generated a clone of Pan02 that was largely resistant to 10 µM of *PCARBTP*), while *PAPTP*-resistant clones could not be obtained, presumably due to the very rapid action of the non-hydrolysable *PAPTP* at the level of mitochondria [9]. We thus compared the protein expression of 3 independent PCARBTP-resistant clones to 3 normal parental Pan02 cells by Sequential Window Acquisition of all Theoretical Mass Spectra (SWATH) nanoLC-MS/MS. Supplementary Figure S2 shows Kv1.3 expression in *PCARBTP* resistant clones. The analysis showed that 50 proteins were consistently upregulated in all three clones and 8 proteins were downregulated (Figure 9A). STRING and pathway enrichment analysis grouped these proteins into several functional classes: a metabolism of xenobiotics by cytochrome P450, an antioxidant defense system, and metabolic pathways of carbohydrates, amino acids and carboxylic acid (Figure 9B). The eight downregulated proteins identified by proteomics (Anxa2, Anxa1, Vcl, Cand1, Cyb5r3, Dpy30, Slc7a5, Dnajc4) were not linked to any enriched pathway. Volcano plot analysis to analyze significant up/down regulated proteins (PCARBTP/Pan02 control) found aldehyde dehydrogenase 3 family member A1 (ALDH3A1), previously shown to confer oxidative stress resistance [22], was the most upregulated protein (Figure 9C). Supplementary Figure S4 shows the antibody specificity of ALDH3A1.

To test the validity of our approach, an ALDH3A1 stable knockdown was created using shRNA via lentiviral transduction (Figure 9D). MTT assay was then performed to determine the sensitivity of ALDH3A1 shRNA and control sHRNA transfected Pan02 cells to *PCARBTP* or to the other drugs used in our study (GEM + Abraxane) (Figure 9E). ALDH3A1 knockdown restored sensitivity in the normal Pan02 cells to the triple treatment with PCARBTP + GEM + Abraxane and also to GEM alone, to GEM+ Abraxane and (Figure 9F), indicating that ALDH3A1 stays at the crossroad of the action of these three drugs.

## 4. Discussion

In the present study, we show that treatment with mitochondrial Kv1.3 inhibitors *PAPTP* and *PCARBTP* in a syngeneic orthotopic mouse model using mouse Pan02 cells resulted in a reduction in tumor size by almost 90% (please check results. The single treatment is not given there). When combined with cytotoxic chemotherapy Gemcitabine and Abraxane, tumor size reduction reached 95%, which is a reduction that has not been observed so far to our knowledge. Most importantly, this treatment strategy did not result in visible organ toxicity. We chose to use Gemcitabine and Abraxane as it is one of the common cytotoxic chemotherapy regimens used in the treatment of PDAC, has been used in the MPACT trial and is a two-drug regimen in comparison to FOLFIRINOX (5-fluorouracil, oxaliplatin, irinotecan), which involves three drugs and associated with different toxicity profile [23,24]. Although we employed Abraxane along with the other inhibitors, in our Pan02 model, the relevance of Abraxane may be lower than in human PDAC, given that in the present animal model stroma is less dense than in the human pathology [15].

We found that Kv1.3 was highly expressed in a large number of resectable human pancreatic cancer specimens (55). This is consistent with previous data from us showing by Affymetrix as well as by Western blots that Kv1.3 is highly expressed in various, largely chemoresistant human PDAC cell lines harboring p53 mutations (PANC-1, AsPC-1, BxPC-3, Capan-1, Colo-357, MiaPaCa2) [9]. The channel is present also in the mitochondrial fraction of these cells [25]. A previous investigation by Bielanska et al. showed that Kv1.3 was under-expressed in human PDAC specimens, but these immunohistochemical studies were performed in a very limited sample (*n* = 2) of patients [26]. Another study examined 18 patients and found a correlation between Kv1.3 expression decrease and metastasis [27]. Importantly, both studies were performed on tumor samples from patients with metastatic PDAC, which is biologically different from those with resectable, non-metastatic disease. Given that our study found that Kv1.3 is highly expressed in resected PDAC, it is possible that during the transition to a metastatic phenotype, pancreas cancer cells are able to down regulate Kv1.3, possibly due to methylation of the promoter region of the *Kcna3* gene encoding for Kv1.3, or another unexplained mechanism [27]. The findings reported here are consistent with previous studies showing that K^+^ channels can promote proliferation [1], while downregulation of Kv1.3 renders cells resistant to apoptotic stimuli [28]. In addition, overexpression of the channel in our patient samples suggests Kv1.3 is a novel therapeutic target to treat pancreas cancer, although the specific role of mitochondrial Kv1.3 in patients with PDAC has not been addressed here. All tumor specimens showed Kv1.3 expression, at least at a low level. Even in patient samples with low Kv1.3 expression, there are some cells that express Kv1.3 and are likely to respond to Kv1.3 inhibitors, although whether these specific cells correspond to cancer cells with specific characteristics or eventually to cancer stem cells remains to be determined.

When examining if Kv1.3 expression holds prognostic value, we found no association with overall or recurrence free survival. It is plausible that in this patient population with resectable PDAC, compared to metastatic PDAC, alterations at the DNA and/or protein level have not occurred to Kv1.3 to render the channel a prognostic biomarker. We also examined if there was an association of Kv1.3 expression with presence of nodal metastases, LVI, and PNI and found there was none. This is likely due to the high rates of PNI (91%), LVI (56%), and positive lymph nodes (84%) in these patients even with localized disease. The predictive value of mitochondrial Kv1.3 expression to *PAPTP* and *PCARBTP* treatment in mice was not tested in this study but we have previously shown a positive correlation between Kv1.3 expression and PCARBTP/PAPTP-induced death in human PDAC lines and between the expression of the channel in mitochondria and the plasma membrane [9].

Mechanistically, the signaling pathway of *PCARBTP* or *PAPTP*-induced tumor cell death was associated with ROS production and the ability of these drugs to drive the cells above a critical ROS level, as indicated by the finding that N-acetyl-cysteine, a molecule able to boost the antioxidant system, prevented the in vivo tumor reducing effect of both drugs [9]. Here, we show that additional mechanisms also come into play, namely an increase in the phosphorylation of p38/MAPK. When treated with the p38 inhibitor, SB203580, there was a reduction in *PCARBTP*-mediated in vivo effect, suggesting that mitoKv1.3 inhibition triggers, at least in part, the p38-mediated death pathway. These findings are consistent with existing data that show activation of the p38/MAPK pathway to be a favorable prognostic marker and associated with improved overall survival in patients with PDAC [29,30,31]. Interestingly, apoptosis induced by a ROS-producing agent (MC3 a Thioredoxin Reductase inhibitor), was also associated with mitochondrial dysfunction and activation of p38-MAPK in a PDAC line. ROS scavengers or inhibition of p38 signaling with SB203580 attenuated MC3-induced apoptosis [32], indicating a relationship between redox balance, p38MAPK activation and cell death in PDAC cells. This connection is further indicated by the finding that p38-MAPK is activated also by GEM [13]. In our case, ROS release is triggered at mitochondria by mitoKv1.3 inhibitors. Given that ROS production due to NOX activation upon GEM treatment [21] has previously been reported, it is reasonable to propose that the synergistic effect of *PAPTP/PCARBTP* and gemcitabine on cell death can be ascribed to an enhanced p38-MAPK activation and downstream signalling and high oxidative stress upon combined treatment.

Despite an unprecedented 95% tumor growth inhibition using a combination of mitoKv1.3 inhibitors and cytotoxic chemotherapies, there were still viable tumor cells. After developing a *PCARBTP* treatment resistant cell line, proteomic analysis revealed there was an almost a four-fold increase in the antioxidant system. Compared to normal human cells, cancer cells have increased ROS generation due to reduced ability to produce scavengers [14,33]. On the other hand, adaptive mechanisms enable cancer cells to escape from oxidative damage by means of overexpressing ROS scavengers. Inhibition of mitoKv1.3 can initiate a cascade of events that leads to transient hyperpolarization of the inner mitochondrial membrane, formation of ROS, stimulation of permeability transition pore, release of cytochrome c, and resulting apoptosis [14,28]. In addition, our recent findings indicate that PAP-1 derivatives bound to mitoKv1.3 are positioned in a way that their psoralenic moieties can directly accept electrons from complex I of the respiratory chain and donate these electrons to molecular oxygen, further boosting ROS [11]. Given the importance of ROS generation to the mechanism of mitoKv1.3 inhibitor mediated cell death, it is not surprising to see that cancer cells resistant to *PCARBTP* upregulated the antioxidant system (GSR, GSTO1, GSTA2, ALDH3A1). An intrinsic resistance to GEM has been linked to upregulation of antioxidant defense system also in AsPc-1 human pancreatic tumor cells and in related orthotopic xenograft mouse model [21]. Indeed, GEM in combination with β-phenylethyl isothiocyanate (PEITC) is able to deplete the cells of glutathione and thus enhance oxidative stress, and it caused the tumor size to reduce to a higher extent than GEM alone. Nonetheless, a reduction of up to 60% occurred in vivo. Thus, even though the principle underlying the effect of GEM along with agents that induce oxidative stress is similar in the two studies (the one by Ju et al. and us), the present paper demonstrates that combination is more efficient when using a drug that is able to release ROS rapidly and to great extent by modulating mitochondrial function. Proteins linked to the cytochrome P450-mediated detoxification system are upregulated (EPHX1, GSTO1, GSTA2), as this system is mainly responsible for xenobiotic metabolism in the cells [34]. In addition, an upregulation of proteins linked to metabolic processes such as glycolysis, carboxylic acid biosynthesis and amino acid synthesis can be observed. This result can be interpreted in light of the inhibition of the mitochondrial respiration and ATP production [9].

In summary, our study indicates that a combination treatment using GEM and mitoKv1.3 inhibitors along with Abraxane represents a promising way to defeat PDAC and opens the possibility to identify further drug combinations that allow complete eradication of the tumor. Importantly, the fact that the pro-oxidant activity of mitoKv1.3 inhibitors is linked to the expression of Kv1.3, offers an important layer of selectivity towards killing of PDAC cells, given that only 8% of normal PDAC specimens showed expression of the channel to a detectable level. Future studies should focus on using mitoKv1.3 inhibitors to selectively kill cells with higher Kv1.3 expression, which may result in not only the direct killing of cancer cells but also enhance the activity of cytotoxic chemotherapies and potential immunotherapies.

## 5. Conclusions

We demonstrated that the potassium channel Kv1.3 is highly expressed in human pancreas adenocarcinoma. MitoKv1.3 inhibitors combined with cytotoxic chemotherapies can result in greater than 95% tumor growth reduction without organ toxicity in a preclinical orthotopic model. SWATH-MS and STRING and pathway enrichment analysis found that the proteins linked to the antioxidant defense system, and the metabolic pathways of carbohydrates, amino acids and carboxylic acid are different in *PCARBTP* resistant clones. These data suggest utilizing Kv1.3 should be considered a novel therapeutic target for pancreatic cancer treatment.

## Data Availability

Data supporting reported results can be provided by the corresponding author upon request.

## Supplementary Materials

The following supporting information can be downloaded at: https://www.mdpi.com/article/10.3390/cancers14112618/s1, Figure S1: Recurrence free and overall survival based on Kv1.3 expression in resected human pancreatic cancer tissues; Figure S2: Western blot analysis showing expression of Kv1.3 in Pan02 wild type and *PCARBTP* resistant cells. β-actin provided as loading control; Figure S3: Immunofluorescence in wild type Pan02 cells showing co-localization of TOM20 and Kv1.3 to the mitochondria; Figure S4: Western blot analysis showing expression and specificity of phospho-p38 and ALDH3A1 antibodies in Pan02 cells; Figure S5: Flow cytometry histograms of the mitoSOX and TMRM analyses.
