# Supplementary material for: Inhibition of a Mitochondrial Potassium Channel in Combination with Gemcitabine and Abraxane Drastically Reduces Pancreatic Ductal Adenocarcinoma in an Immunocompetent Orthotopic Murine Model
Source: Cancers (Basel). 2022 May 25;14(11):2618. doi: 10.3390/cancers14112618 (PMC9179813; doi:10.3390/cancers14112618)

Original WB for Figure 7

P-p38 MAPK(Thr180/Tyr182)

β-actin

PCARBTP (μM) 0 0.1 0.5 1 10 100

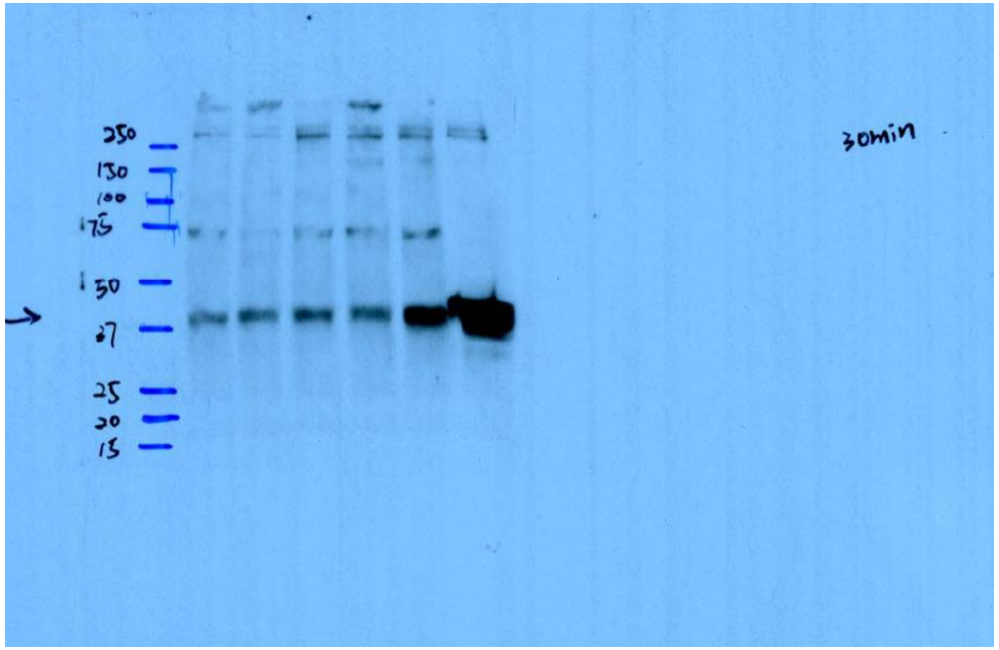

PCARBTP (μM) 0 0.1 0.5 1 10 100

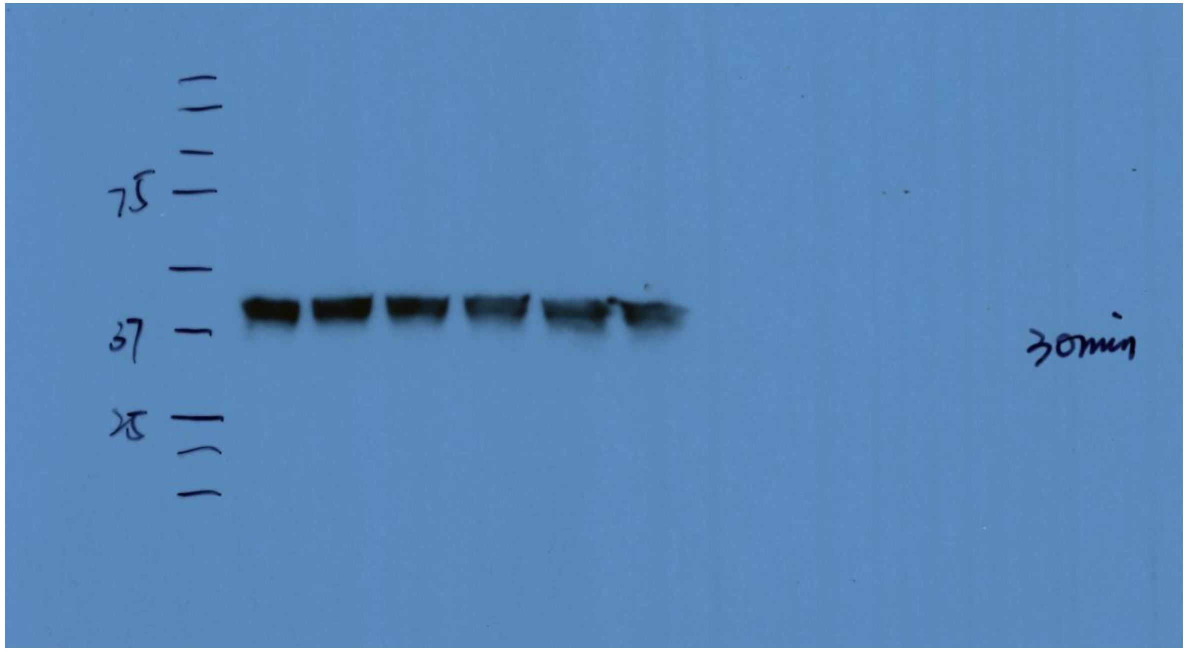

Original WB for Figure 9

ALDH3A1

CTL shRNA | ALDH3A1 shRNA

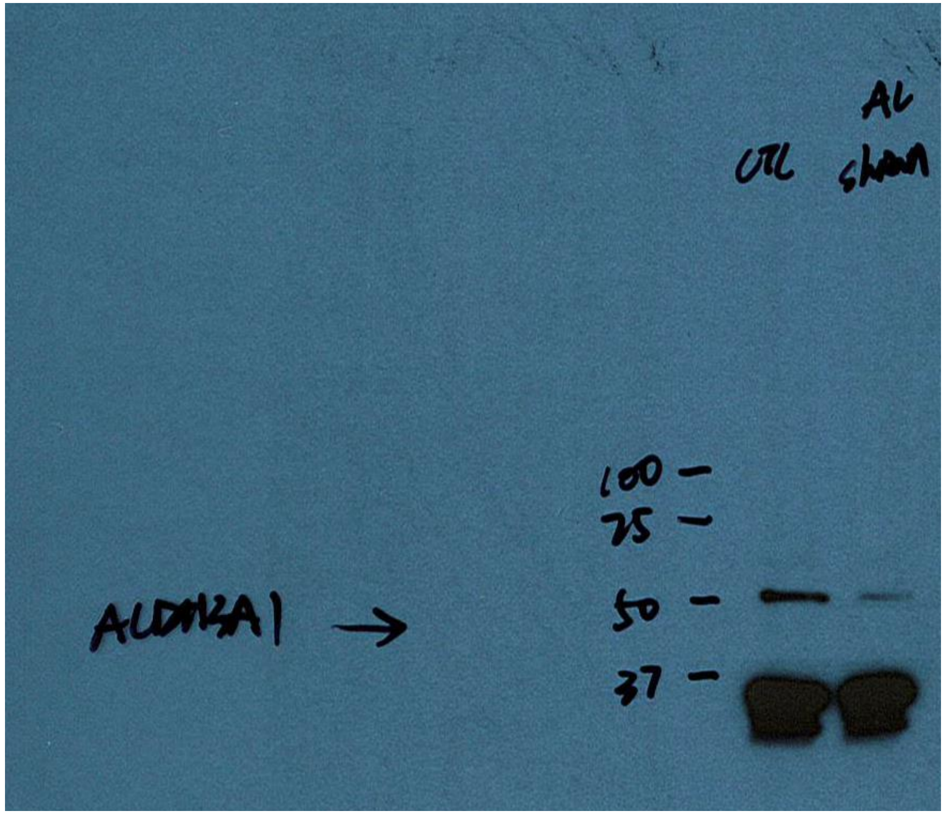

β-actin

CTL shRNA | ALDH3A1 shRNA

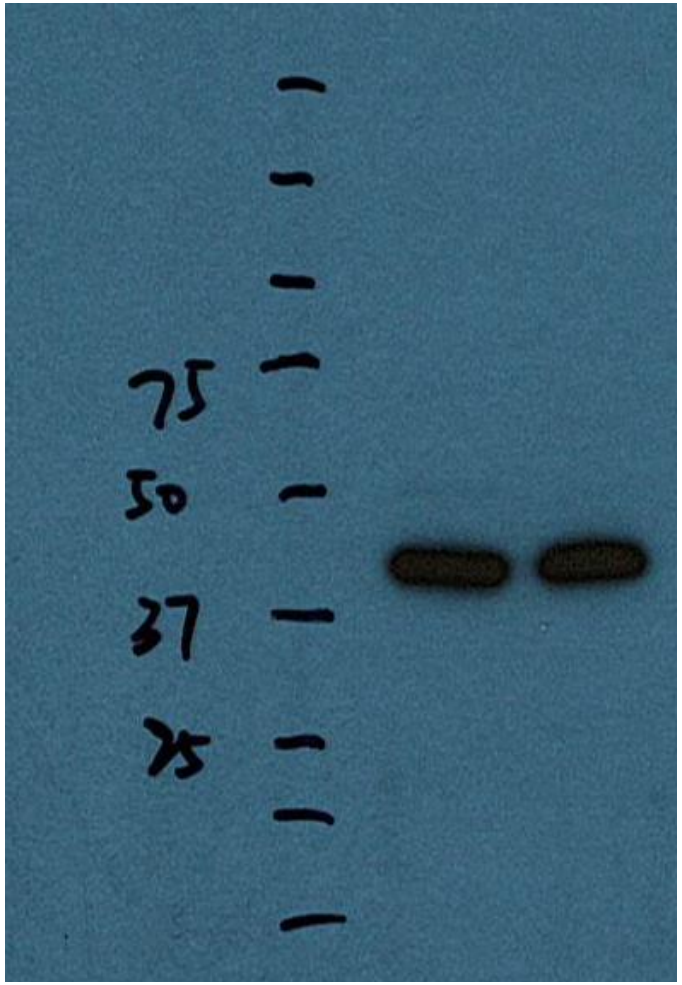

Supplement: Supplementary file 1 [file cancers-14-02618-s001.zip › cancers-1705530-supplementary/cancers-1705530 Original Western Figure 7 and Figure 9.pdf]
