# Supplementary material for: Inhibition of a Mitochondrial Potassium Channel in Combination with Gemcitabine and Abraxane Drastically Reduces Pancreatic Ductal Adenocarcinoma in an Immunocompetent Orthotopic Murine Model
Source: Cancers (Basel). 2022 May 25;14(11):2618. doi: 10.3390/cancers14112618 (PMC9179813; doi:10.3390/cancers14112618)
Supplement: Supplementary file 1 [file cancers-14-02618-s001.zip › cancers-1705530-supplementary/cancers-1705530-supplementary.pdf]

cancers
MDPI

Supplementary Materials

Inhibition of a Mitochondrial Potassium Channel in Combination with Gemcitabine and Abraxane Drastically Reduces Pancreatic Ductal Adenocarcinoma in an Immunocompetent Orthotopic Murine Model

Weiwei Li, Gregory C. Wilson, Magdalena Bachmann, Jiang Wang, Andrea Mattarei, Cristina Paradisi, Michael J. Edwards, Ildiko Szabo, Erich Gulbins, Syed A. Ahmad and Sameer H. Patel

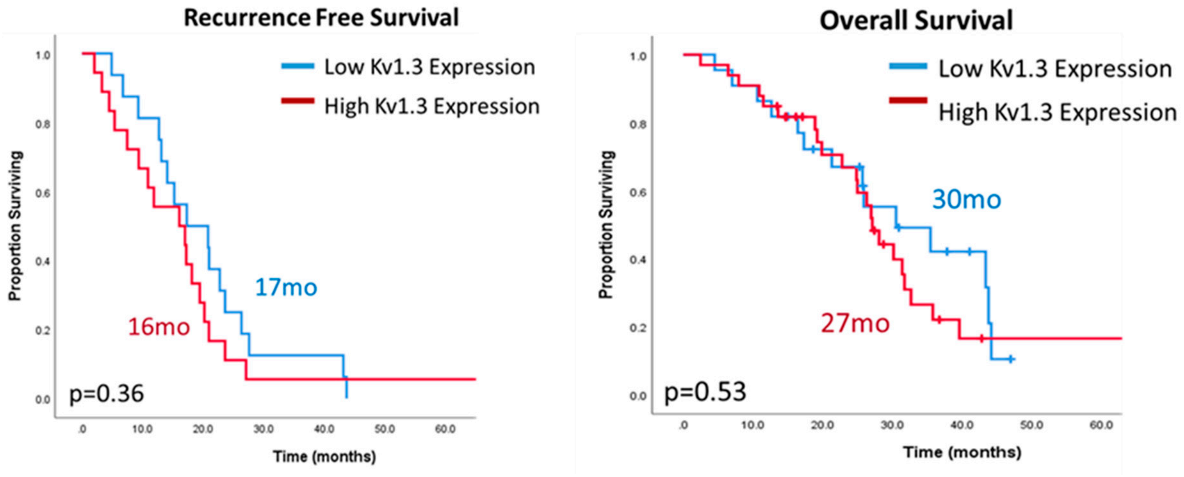

Figure S1. Left: Recurrence free survival and overall survival (right) based on Kv1.3 expression in resected human pancreatic cancer tissues.

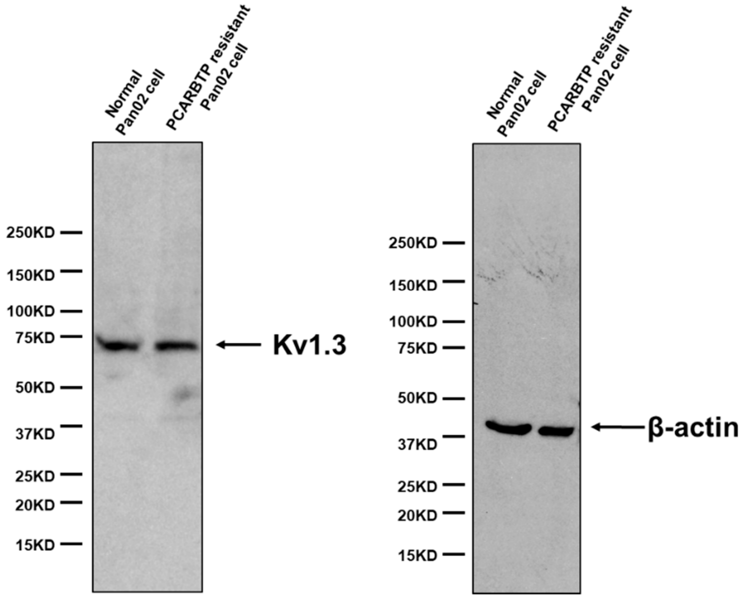

Figure S2. Western blot analysis showing expression of Kv1.3 in Pan02 wild type and *PCARBTP* resistant cells. β-actin provided as loading control.

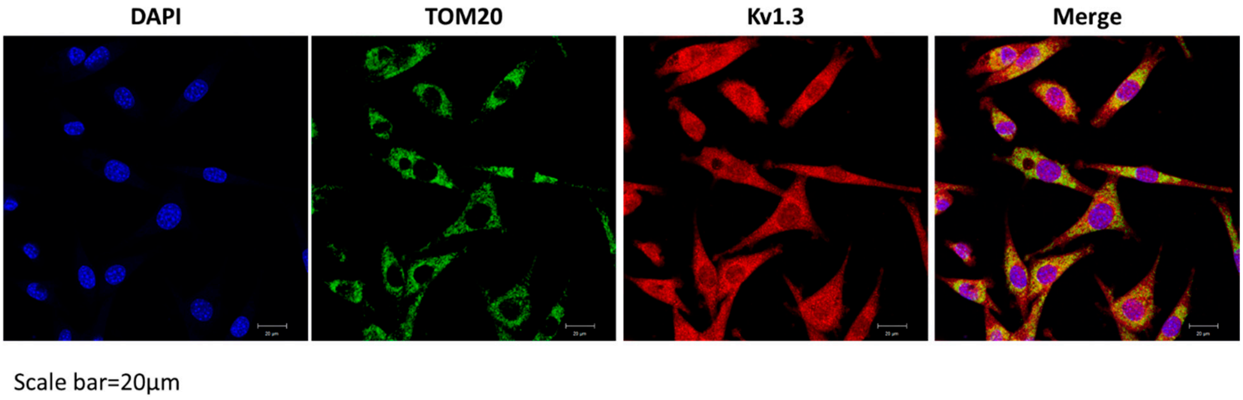

Figure S3. Immunofluorescence in wild type Pan02 cells showing co-localization of TOM20 (green) and Kv1.3 (red) to the mitochondria. The co-localization coefficient of TOM20 and Kv1.3 was 0.82. Scale bar represents 20μm.

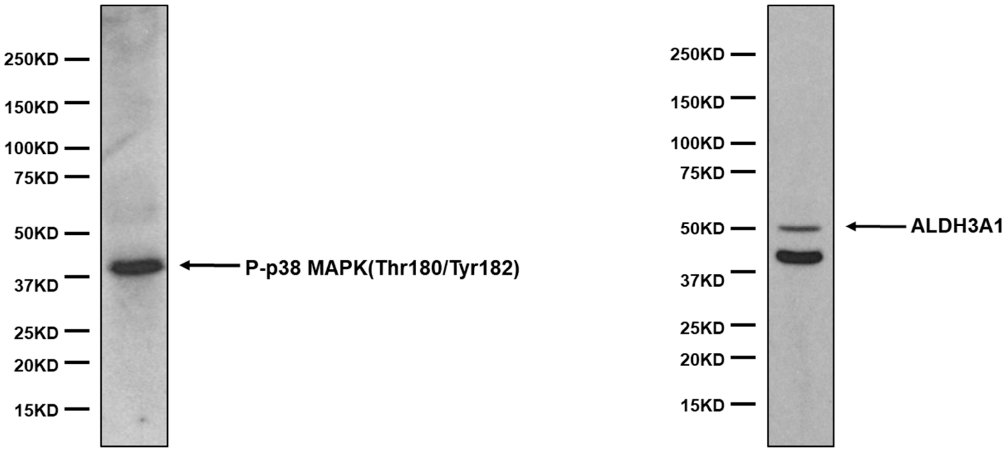

Figure S4. Western blot analysis showing expression and specificity of phospho-p38 and ALDH3A1 antibodies in Pan02 cells. P-p38 MAPK (1: 1000; Cell signalingCST, MA, USA)**.**

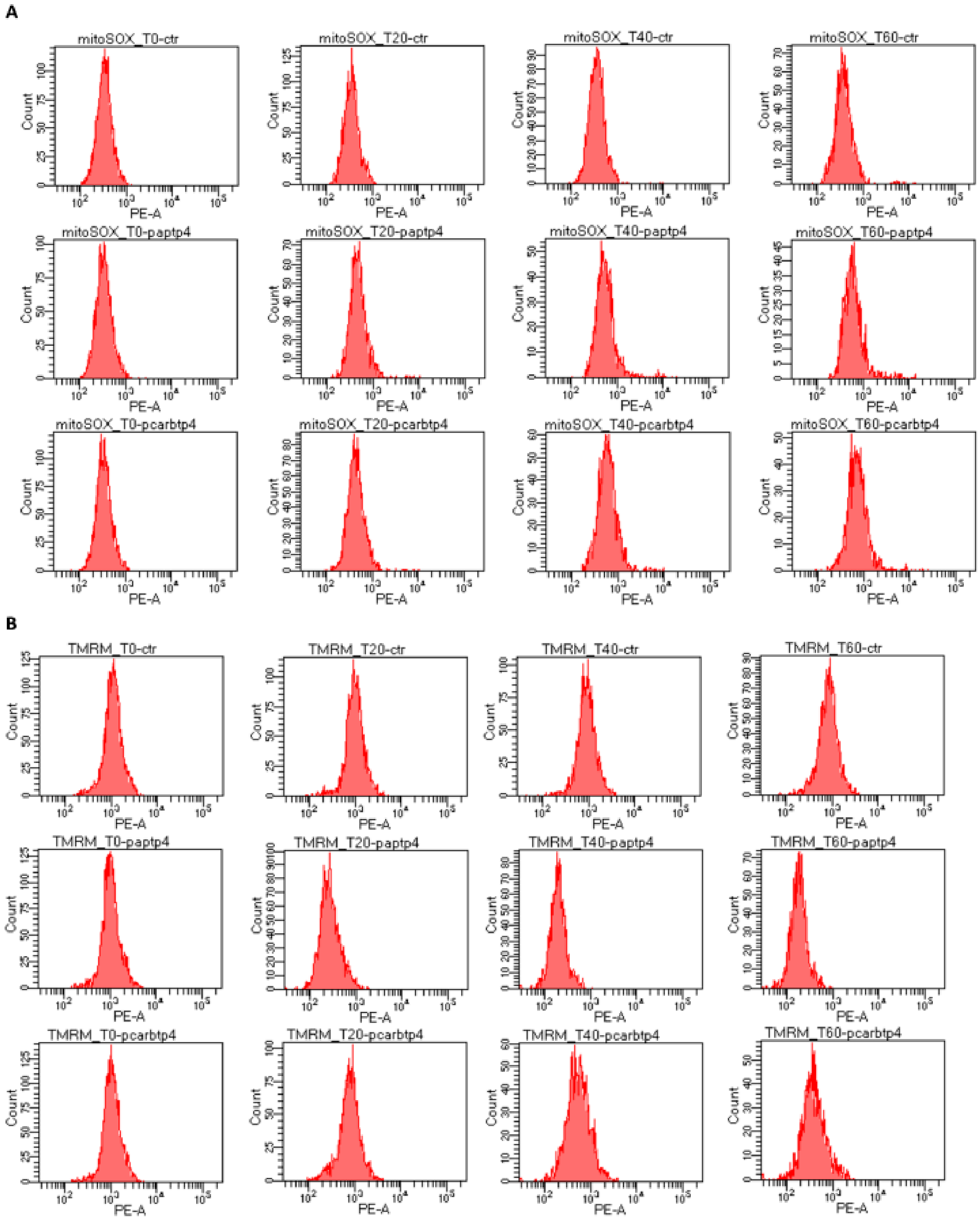

Figure S5. Flow cytometry histograms of the mitoSOX (A) and TMRM analyses.
