# Supplementary figures and images for: Inhibition of a Mitochondrial Potassium Channel in Combination with Gemcitabine and Abraxane Drastically Reduces Pancreatic Ductal Adenocarcinoma in an Immunocompetent Orthotopic Murine Model
Source: Cancers (Basel). 2022 May 25;14(11):2618. doi: 10.3390/cancers14112618 (PMC9179813; doi:10.3390/cancers14112618)

Original Western Blot For Figure S3

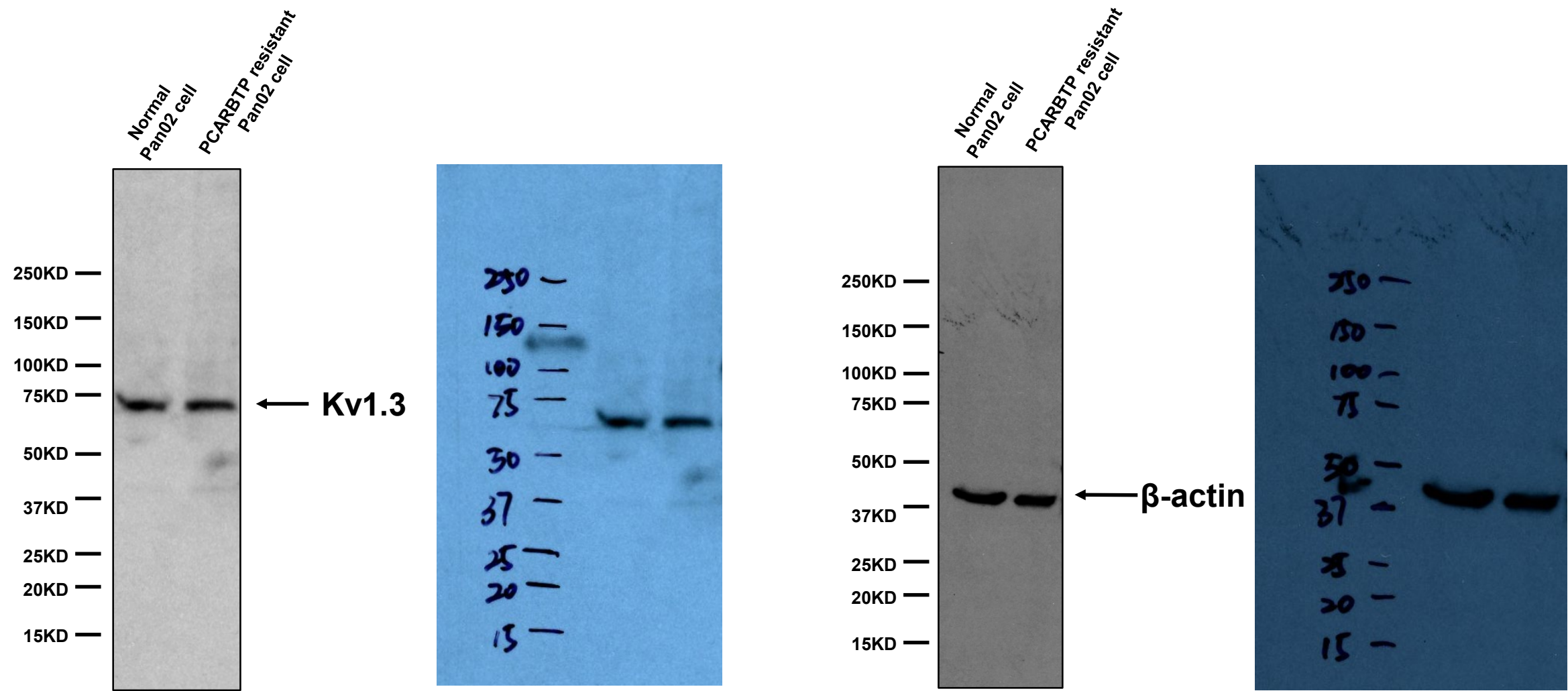

Original Western Blot For Figure S4

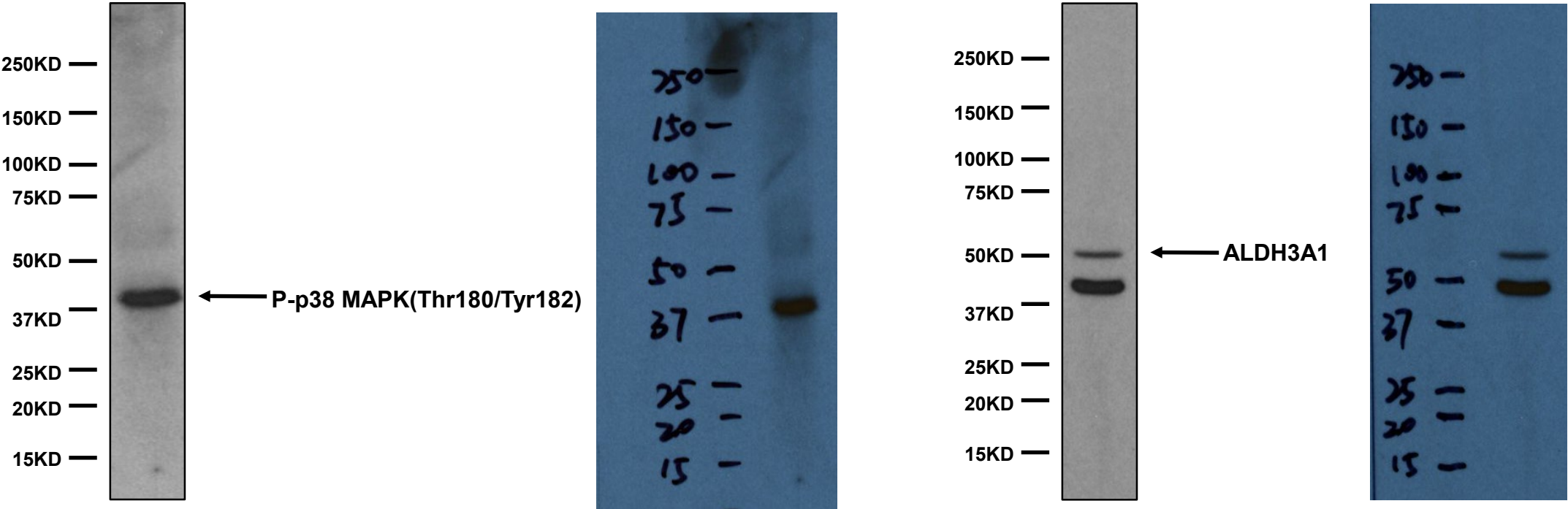

Supplement: Supplementary file 1 [file cancers-14-02618-s001.zip › cancers-1705530-supplementary/cancers-1705530 - Original Western Blot S3 and S4.pdf]
